# Kidney adiposity in obesity and chronic kidney disease: mechanisms, measurement, and clinical implications

**DOI:** 10.3389/fmed.2026.1967640

**Published:** 2026-10-22

**Authors:** Yunxi Zhang

**Affiliations:** School of Medicine, Tulane University, New Orleans, LA, United States

**Keywords:** adipose-kidney crosstalk, chronic kidney disease, ectopic renal lipid, imaging, obesity, perirenal adipose tissue, renal sinus fat, treatment associated remodeling

## Abstract

Kidney outcomes vary substantially among people with similar body mass index (BMI), and conventional measures of obesity do not specify the adipose compartments that lie closest to the kidney or the biological state of those tissues. Human imaging, physiological studies, and paired-tissue analyses now permit a more anatomically explicit account of kidney adiposity. Perirenal adipose tissue (PRAT), renal sinus adipose tissue (RSAT), and intrarenal lipid are distinct constructs and should not be collapsed into a single ‘fatty kidney’ construct. This critical narrative review examines kidney adiposity at the obesity–CKD interface using a claim-conditioned translational evidence continuum spanning association, partial independence from systemic adiposity, prospective association, human mechanistic support, treatment-associated depot remodeling, depot–renal response linkage, causal mediation, and treatment-effect modification. Selected kidney-associated fat measures are associated with albuminuria, filtration, intrarenal haemodynamics, and CKD status, and some signals persist after adjustment for BMI or visceral adipose tissue. In paired tissue from healthy kidney donors, PRAT and RSAT show distinct metabolic and immune profiles. However, much of the human evidence derives from cohorts enriched for type 2 diabetes, which limits extrapolation to non-diabetic obesity-associated CKD. Acquisition, segmentation, normalisation, external validation, and clinically useful thresholds also remain unsettled. Intervention studies show that selected kidney-associated fat measures can change with lifestyle or sodium-glucose cotransporter-2 inhibition, whereas major kidney-outcome trials often did not measure kidney-adiposity compartments. These parallel observations do not establish that depot remodeling mediates renal protection. Compartment-resolved assessment of kidney adiposity should therefore be regarded as a research strategy rather than a clinical classification. Its future value depends on incremental prognostic or decision information beyond eGFR, albuminuria, and validated CKD risk models, and on direct testing of whether depot modification contributes causally to kidney response.

## 1 Introduction: from body size to kidney-specific adiposity

Obesity and CKD frequently coexist, but the clinical relationship between them is less uniform than a BMI-based description implies. Two patients with the same BMI can differ substantially in visceral adiposity, ectopic lipid storage, insulin resistance, blood pressure, albuminuria, and the trajectory of kidney function. Recent attempts to refine the diagnosis of obesity have therefore shifted emphasis from body size alone toward excess adiposity, fat distribution, and organ-level consequences (1). In CKD, this problem is amplified by fluid retention, sarcopenia, age-related changes in body composition, and the high prevalence of diabetes and cardiovascular disease; all can weaken the biological meaning of BMI at the individual level (2). These limitations justify moving beyond a single anthropometric measure, but they do not by themselves establish what should replace it.

For the kidney, the relevant exposure is unlikely to be captured by total adiposity alone. Visceral fat contributes to systemic insulin resistance, inflammation, neurohumoral activation, and hypertension, and genetic epidemiology supports a relationship between visceral adiposity and CKD risk (3, 4). Yet the kidney also sits within a local adipose environment. Perirenal fat surrounds the kidney outside the renal capsule, renal sinus fat occupies a confined compartment contiguous with renal vessels, lymphatics, and the collecting system, and lipid can accumulate within renal parenchymal cells. These compartments differ in anatomy, potential mechanisms, and measurement (Table 1). A high visceral-fat burden therefore provides metabolic context, whereas kidney-adjacent or intrarenal fat may describe a more proximal renal exposure. The distinction matters because a biomarker that is merely correlated with visceral obesity will add little to clinical assessment, while a kidney-specific measure could be informative if it captures renal risk, mechanism, or treatment response not otherwise apparent.

**Table 1:** Anatomical and methodological definition of kidney-adiposity compartments.

Domain/compartment	Anatomical or biological construct	Main modality	Candidate metric	What it may capture	Major limitation
Systemic adiposity context	Whole-body or central adiposity; not kidney-specific	Anthropometry; CT/MRI	BMI, waist circumference, waist-to-height ratio, VAT area/volume	Background metabolic burden and confounding context	BMI is insensitive to distribution; VAT is not renal-specific
Perirenal adipose tissue (PRAT)	Adipose tissue surrounding the kidney outside the renal capsule	Ultrasound; CT; MRI	Posterior thickness, area, volume, attenuation	Regional kidney-adjacent adiposity; possible local/metabolic exposure	Landmarks and inclusion of pararenal layers vary across studies
Renal sinus adipose tissue (RSAT)	Adipose tissue within the renal sinus adjacent to vessels, lymphatics and collecting structures; imaging studies often quantify this depot as renal sinus fat (RSF).	CT; MRI	Area, volume, percentage/fraction	Confined local depot with potential haemodynamic/mechanical relevance	Segmentation and normalisation not standardised
Intrarenal/parenchymal lipid	Lipid within renal tissue rather than surrounding adipose tissue	Dixon MRI; MRS/MRSI	Renal proton-density fat fraction or spectroscopic lipid signal	Ectopic renal lipid burden	Imaging does not identify cell type or toxic lipid species
Adipose tissue quality	Biological state not captured by volume alone	CT; MRI; tissue assays	CT attenuation, MRI composition, transcriptomic/inflammatory signatures	Inflammation, fibrosis or metabolic function hypotheses	No validated imaging-to-tissue calibration for routine CKD care
Renal response layer	Organ response linked to exposure rather than adiposity itself	Laboratory; physiology; imaging	UACR, measured/estimated GFR, renal vascular resistance, eGFR trajectory	Whether adiposity is biologically relevant to the kidney	Must not be folded into an adiposity score without validation

No clinical cut point is proposed. Metrics are research measures whose interpretation depends on modality and population.

The literature has progressed faster than the terminology. Broad reviews have already detailed haemodynamic, endocrine, inflammatory, neural, and lipotoxic pathways linking obesity to kidney injury (3, 5, 6). A systematic review has separately summarised the association between perirenal fat and CKD (7), while more recent work has examined depot-specific adipose biology, clinical assessment, and therapeutic remodeling across metabolic disease (8). Contemporary 2026 reviews further frame obesity diagnosis and management in CKD from a clinical perspective (9) and renal parenchymal lipid dysregulation and fibrosis under the broader concept of fatty kidney disease (10). These syntheses reinforce the importance of adiposity and renal lipid biology but do not resolve the translational question addressed here. Repeating pathway- or depot-centred summaries would add little. The unresolved question is different: when does a kidney-fat measurement remain an anatomical correlate of systemic adiposity, and what additional evidence is required before it can be interpreted as a mechanistically informative marker or a treatment-responsive clinical marker?.

Accordingly, this review is organised by the claim a study design can support rather than by signalling pathway, adipose depot, or drug class. We first define the anatomical and methodological components of kidney-adiposity assessment, then ask whether regional measures add information beyond systemic adiposity, whether prospective and human mechanistic evidence strengthens that interpretation, and whether intervention-associated depot change can be linked to a renal response. The therapeutic section deliberately separates studies that directly measured kidney-adiposity change from large kidney-outcome trials that did not measure these compartments. Improvement in kidney outcomes and reduction of kidney-associated fat can coexist without proving that one mediates the other. The compartments described here are therefore treated as research measurements rather than as a proposed clinical classification, score, or treatment-selection system.

## 2 Literature identification and evidence framing

This is a critical narrative review rather than a systematic review. PubMed was used as the principal bibliographic source, with targeted searches through 31 August 2026 for combinations of obesity, CKD, perirenal or perinephric adipose tissue, renal sinus fat, renal ectopic lipid, computed tomography, magnetic resonance imaging, ultrasound, renal haemodynamics, adipose transcriptomics, SGLT2 inhibition, GLP-1 receptor agonism, weight loss, bariatric or metabolic surgery, automated segmentation, deep learning, and risk prediction. The PubMed search was supplemented by backward and forward citation tracking and related-article searches. The search was designed to identify literature needed for critical synthesis rather than to provide exhaustive coverage across bibliographic databases and is therefore not presented as a systematic search. Priority was given to original human studies, prospective cohorts, randomized trials, direct human tissue or physiological investigations, high-quality systematic reviews, and contemporary consensus documents. Diabetes-enriched cohorts were retained because much of the renal-fat literature originates in type 2 diabetes, but their evidentiary context is identified rather than treated as representative of all patients at the obesity–CKD interface. Generative AI assistance was used during manuscript preparation: ChatGPT (OpenAI; model GPT-5.6 Sol; web application accessed August 2026) supported language editing, evidence organisation, and preparation or refinement of schematic figures. The author independently verified the scientific claims, numerical values, citations, and final figures and takes responsibility for the content of the manuscript. A study-level evidence map of the human studies used to structure the synthesis is provided in Supplementary Table S1.

For interpretation, anatomical definition and measurement validity were treated as prerequisites because a depot-specific claim is difficult to interpret when the compartment or protocol is unclear. Studies were then compared using a consistent set of appraisal domains: anatomical specificity, measurement reproducibility, population spectrum, cross-sectional vs. longitudinal design, adjustment for systemic adiposity and established renal risk markers, endpoint type, sample size and event count, external validation, and whether null or discordant findings were reported. Studies described as representative were selected to illustrate the strongest or most informative human evidence for each type of claim while preserving variation in depot, modality, population, and direction of findings; selection was not based on statistical significance. The literature was organised along a claim-conditioned translational evidence continuum rather than assigned a formal quality grade. Cross-sectional studies can establish association; analyses accounting for BMI or visceral adiposity address partial non-redundancy; prospective cohorts add temporal or prognostic information; and human haemodynamic, tissue, or functional-genomic studies strengthen mechanistic plausibility. Intervention studies demonstrate treatment-associated depot remodeling only when a kidney-associated compartment is measured longitudinally. Causal mediation and treatment-effect modification require stronger designs and analyses. Large kidney-outcome trials without depot measurement provide therapeutic context but do not advance the depot-specific evidence continuum, whereas design-only mechanistic trials are treated as planned bridges rather than outcome evidence. Figure 1 summarises this continuum, Table 2 makes study strengths and limitations explicit, and Supplementary Table S1 provides the study-level evidence map.

**Figure 1:**
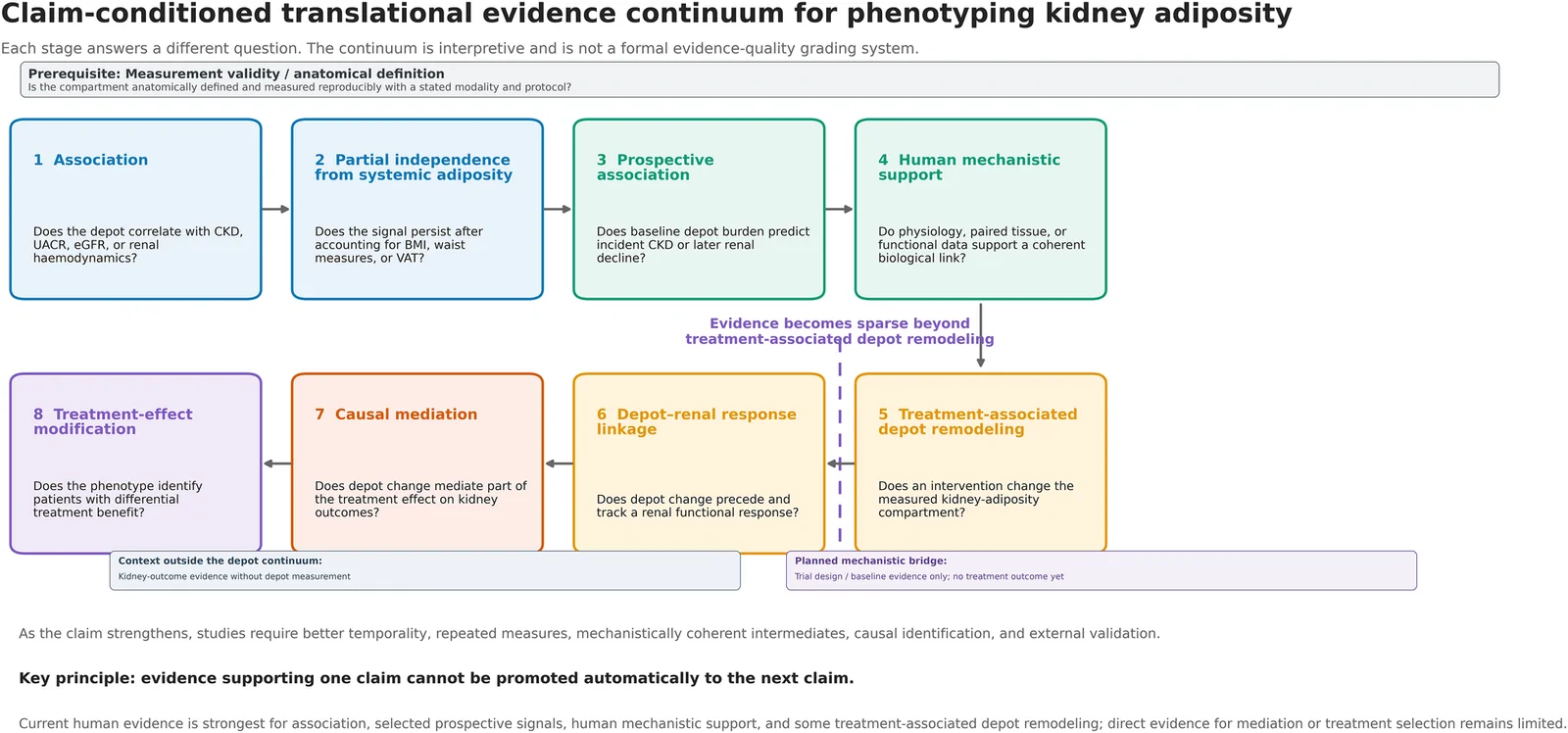
Claim-conditioned translational evidence continuum for kidney-adiposity research. Anatomical definition and measurement validity are treated as a prerequisite before a depot-specific claim enters the continuum. Different study designs then address different questions: association, partial independence from systemic adiposity, prospective association, human mechanistic support, treatment-associated depot remodeling, depot–renal response linkage, causal mediation, and treatment-effect modification. Kidney-outcome trials that do not measure kidney-associated depots provide contextual therapeutic evidence rather than advancement along the depot continuum; design-only mechanistic trials are labelled as planned bridges. Human evidence is strongest for the earlier stages and becomes sparse beyond treatment-associated depot remodeling. The continuum is interpretive and does not constitute a formal evidence-quality grading system

**Table 2:** Representative human studies across the translational evidence continuum for kidney adiposity.

Evidence contribution (Figure 1 continuum)	Study	Population/design	Depot/method	Renal endpoint	Principal inference	Main limitation
Partial independence from systemic adiposity	Foster 2011	Framingham; *n* = 2,923; cross-sectional	RSF by CT	CKD; hypertension	RSF associated with CKD after BMI/VAT adjustment	No temporality
Partial independence from systemic adiposity	Lamacchia 2011	T2D; *n* = 151; cross-sectional	Para/perirenal fat by US	CKD; renal resistance index	Regional fat linked to CKD and renal resistance	Diabetes-specific; cross-sectional
Association	D'Marco 2019	Established CKD; cross-sectional	PRAT by US	Metabolic/renal risk profile	PRAT relevant within CKD population	No longitudinal renal outcome
Human mechanistic support	Spit 2020	T2D; *n* = 51; physiological cross-sectional	RSF by MRI	Measured GFR, ERPF, renal vascular resistance	Human haemodynamic plausibility	Small; cross-sectional
Prospective association	Chen 2021	T2D without CKD at baseline; *n* = 190, 29 incident CKD	PRAT thickness	Incident CKD over 2 y	Prospective temporal signal; HR 1.67 per SD	Few events; no external validation
Prospective association	Fujioka 2023	CKD biopsy cohort; *n* = 56; prospective	RSF accumulation	>50% eGFR decline	Prognostic signal in established CKD	Very small selected cohort
Measurement validity/anatomical definition	Tang 2024	Obesity *n* = 56 + controls *n* = 47	Dixon MRI: renal PDFF, RSF, PRAT	eGFR/early kidney injury	Different renal fat compartments are non-redundant	Modest sample; limited longitudinal outcomes
Human mechanistic support	Kaneko 2024	Biopsy-evaluated CKD; *n* = 132	RSF imaging	Intrarenal haemodynamics; eGFR	RSF signal persisted beyond VAT	Cross-sectional selected cohort
Human mechanistic support	Chen 2024	T2D; *n* = 91; physiological cross-sectional	PRAT + renal physiology	GFR; vascular/afferent resistance	Statistical mediation supports a haemodynamic pathway	Model-derived mediator; not causal
Human mechanistic support	Pereira 2025	Healthy kidney donors; 40 paired depots	PRAT vs. RSAT tissue, RNA-seq, functional assays	Tissue biology	Direct human evidence of depot-specific biology	Healthy donors; not disease causality
Prospective association	Kukla 2026	Living kidney donors; *n* = 3,226	Posterior PRAT by CT + biopsy	Histology; long-term low eGFR	Anatomy-specific signal linked to tissue and outcome	Healthy-donor selection
Association	Peng 2026	Controls + T2D with/without DKD; CT study	Renal/PRAT/RSF quantity and attenuation	DKD status	Fat quality and quantity may provide different information	Retrospective diabetes-focused cohort
Prospective/incremental association	Fan 2025	T2D; *n* = 404; kidney-function follow-up *n* = 125	Perirenal adipose-tissue area/index + CT attenuation	KDIGO risk category; rapid DKD progression	CT perirenal-fat characteristics provided incremental prognostic information in a T2D cohort	Retrospective; diabetes-specific; small longitudinal subset; no external decision-impact validation

The table presents representative studies spanning major anatomical compartments, population contexts, and types of translational claim and is not intended to be exhaustive. RSAT denotes the anatomical renal-sinus adipose depot, whereas RSF is retained when a source study reports an imaging-derived renal sinus fat measure. CKD, chronic kidney disease; CT, computed tomography; eGFR, estimated glomerular filtration rate; ERPF, effective renal plasma flow; MRI, magnetic resonance imaging; PRAT, perirenal adipose tissue; RSAT, renal sinus adipose tissue; RSF, renal sinus fat; T2D, type 2 diabetes; US, ultrasound; VAT, visceral adipose tissue. The broader human evidence map is provided in Supplementary Table S1.

## 3 Kidney-adiposity compartments and biological distinctions

Kidney-associated adipose tissues should not be collapsed into a generic category of ‘fat around the kidney’. Perirenal adipose tissue (PRAT) occupies the retroperitoneal space surrounding the kidney, whereas renal sinus adipose tissue (RSAT) resides within the renal sinus, a restricted anatomical compartment through which the renal vessels, lymphatics, nerves, and collecting structures pass. Intrarenal or renal parenchymal lipid refers to lipid within renal tissue rather than adipose tissue surrounding it. Visceral adipose tissue (VAT), in turn, provides a systemic metabolic context but is not kidney-specific. These distinctions are more than semantic. An imaging thickness measure of PRAT, an MRI-derived renal sinus fat (RSF) volume, and a Dixon-based renal fat fraction sample different biological constructs and should not be pooled conceptually without qualification (11). In this review, RSAT denotes the anatomical depot, whereas RSF is retained when referring to an imaging-derived measure reported by an individual study.

The strongest direct evidence that anatomical labels correspond to biological differences comes from paired human tissue. In healthy kidney donors, Pereira and colleagues compared PRAT and RSAT from the same individuals and found marked depot-specific differences in transcriptomic, metabolic, and inflammatory profiles. PRAT showed greater thermogenic and oxidative signatures and greater insulin-stimulated glucose uptake, whereas RSAT was relatively enriched for macrophage and inflammatory programs (12). These findings argue against treating all kidney-associated fat as uniformly pathogenic. They also caution against the common assumption that more PRAT necessarily means more local inflammation. The study was performed in healthy donors, however, so the direction and magnitude of these depot differences in obesity-associated CKD remain uncertain.

For descriptive purposes, the literature can be organised across four non-exclusive domains (Figure 2). The first is systemic adiposity context, including BMI, waist-based measures, subcutaneous fat, and VAT. The second is kidney-associated anatomical burden, including PRAT, RSAT, and intrarenal lipid. The third is tissue quality or function: CT attenuation, MRI fat fraction, inflammatory or metabolic signatures, and, where available, tissue-level cellular states. The fourth is renal response, expressed through hyperfiltration or reduced filtration, albuminuria, renal vascular resistance, and longitudinal eGFR. This grouping is descriptive and is not a proposed diagnostic or clinical classification. It asks whether the same anatomical fat burden has different implications according to tissue state and renal response, and whether kidney-associated measurements retain information after systemic adiposity is accounted for Key anatomical definitions, imaging modalities, candidate metrics and major limitations for kidney-adiposity compartments are summarised in Table 1.

**Figure 2:**
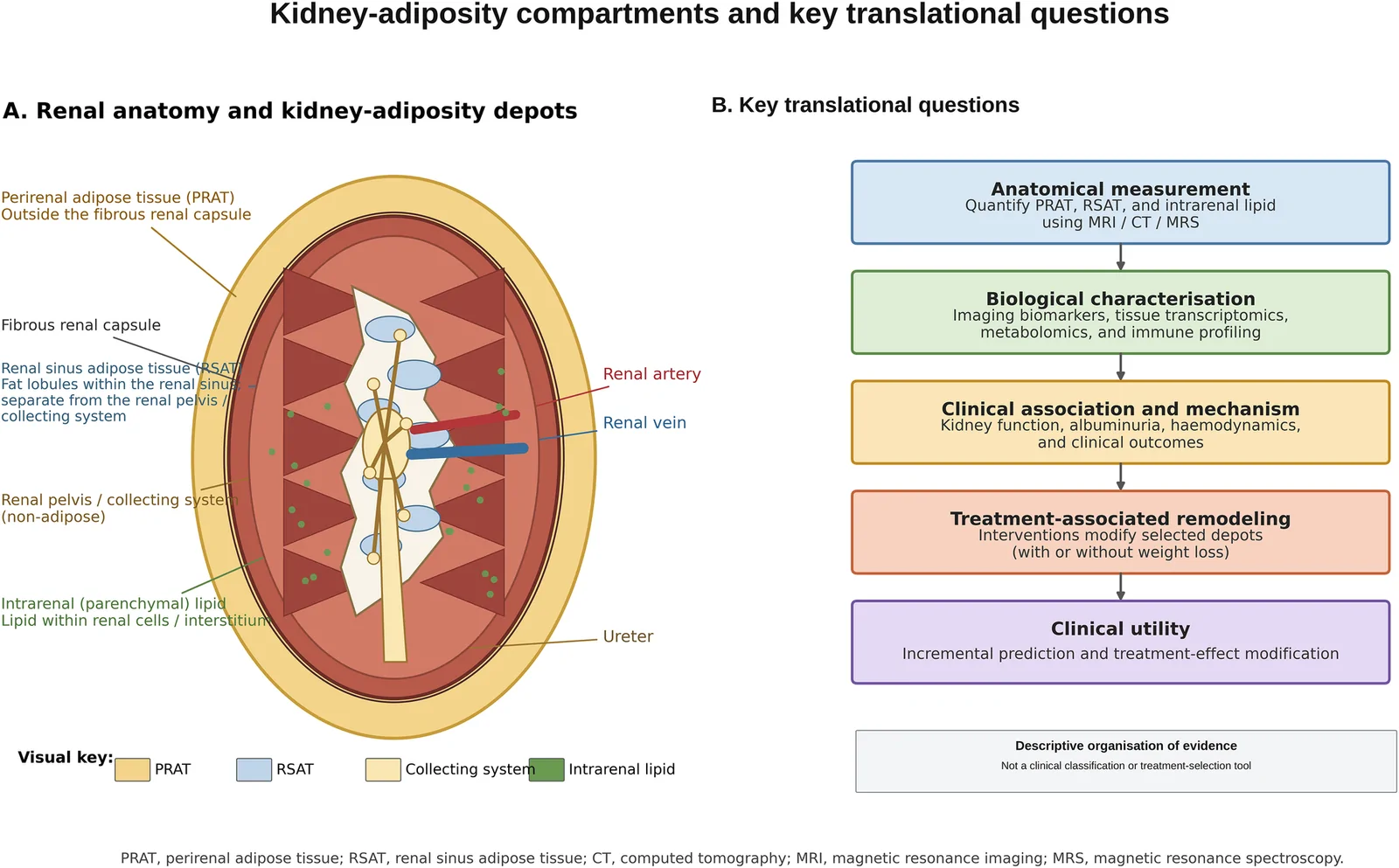
Kidney-adiposity compartments and key translational questions. **Panel A** schematically distinguishes perirenal adipose tissue (PRAT), renal sinus adipose tissue (RSAT), and intrarenal/parenchymal lipid; RSAT is depicted within the renal sinus as adipose tissue distinct from the renal pelvis/collecting system. These compartments occupy different anatomical locations and should not be used interchangeably. **Panel B** links anatomical measurement to biological characterisation, renal association and mechanism, treatment-associated depot remodeling, and clinical utility. The sequence is presented descriptively and is not a validated clinical classification or treatment-selection tool. BMI, body mass index; CT, computed tomography; MRI, magnetic resonance imaging; MRS, magnetic resonance spectroscopy; PRAT, perirenal adipose tissue; RSAT, renal sinus adipose tissue; UACR, urinary albumin-to-creatinine ratio; eGFR, estimated glomerular filtration rate.

## 4 Measuring kidney adiposity: from anatomical visualisation to research quantification

The measurement literature is sufficiently mature to show feasibility, but not sufficiently standardised to support routine clinical thresholds. This distinction is easy to lose. A technique can be reproducible within a research protocol while still lacking anatomical harmonisation, external calibration, or evidence that the resulting value changes a clinical decision. Recent systematic reviews of ultrasound and cross-sectional imaging illustrate this gap: kidney-adjacent fat can be quantified by several approaches, yet landmark selection, segmentation, normalisation, and reporting vary substantially (11, 13).

Ultrasound is the most accessible approach to PRAT. It avoids radiation, is inexpensive, and can be repeated, making it attractive for longitudinal studies and settings without advanced imaging. The trade-off is dependence on operator technique and anatomical landmarks. Studies have variously measured posterior perirenal fat, para-perirenal thickness, or combined layers at different renal levels. A threshold derived in one metabolic or diabetic cohort should not be transferred to another population as a CKD risk threshold. The current literature supports ultrasound as a research measurement platform, not a universal diagnostic test (13). In practical terms, future ultrasound studies should report the exact side, imaging plane, renal landmark, whether the pararenal layer is included, the number of repeated measurements, and inter- or intra-reader reproducibility.

CT offers an anatomical advantage. Thickness, area, and volume can be measured, renal sinus fat can be segmented separately from PRAT, and adipose attenuation can provide a crude signal of tissue composition beyond quantity. Existing abdominal CT obtained for unrelated clinical reasons also raises the possibility of opportunistic assessment. That opportunity should be treated cautiously. CT measures are affected by acquisition parameters, contrast phase, segmentation method, and whether values are indexed to kidney size, body surface area, or total adiposity. Moreover, a statistically different attenuation value between CKD groups is not automatically a clinically interpretable marker of adipose ‘quality’. The recent CT literature in diabetic kidney disease is moving toward combined quantity and attenuation measures, but prospective external validation remains sparse (14). Automated segmentation could reduce manual burden and improve reproducibility in opportunistic CT or MRI workflows. Deep-learning pipelines have demonstrated technically feasible automated kidney and visceral-fat segmentation with PRAT feature extraction in a multicentre renal-oncology cohort, but this does not validate automated kidney-adiposity reporting for CKD; CKD-specific external validation, protocol harmonisation, and clinically acceptable segmentation-error thresholds remain necessary (15).

MRI provides the most versatile research platform because it can separate compartments without ionising radiation and can combine anatomy with tissue composition and functional measures. Renal sinus fat volume, PRAT thickness or volume, and renal parenchymal fat fraction can be quantified within the same examination. Dixon-based techniques have shown that these measures are not redundant; in people with obesity, different renal fat compartments relate differently to early kidney abnormalities, and PRAT can retain an association with eGFR after adjustment for BMI and other fat compartments (16). MRI can also be paired with oxygenation or perfusion-sensitive sequences, which is useful when the aim is to test a physiological pathway rather than simply to classify fat burden. Its disadvantages are cost, protocol dependence, segmentation burden, and reduced feasibility at population scale.

Normalisation deserves more attention than it usually receives. Absolute millimetres of PRAT may partly reflect overall body size; absolute RSAT volume may partly reflect kidney size. Indexing can reduce one source of variation while introducing another. There is no established denominator that converts kidney-associated fat into a universally interpretable renal-risk quantity. Sex, age, ethnicity, diabetes status, and CKD stage may alter both depot distribution and the meaning of a given measurement. Accordingly, a future consensus should specify anatomical nomenclature and acquisition standards before attempting universal cut points. Kidney-associated adiposity can currently be quantified in research settings, but no validated threshold has been established at which treatment, monitoring, or prognostic assessment should change.

## 5 Associations with renal markers and clinical outcomes

### 5.1 Intermediate markers and CKD status

Human association studies consistently suggest that kidney-associated fat is not merely an imaging curiosity. The Framingham Heart Study provided an early population-level signal: renal sinus fat was associated with hypertension and CKD, and the association with CKD persisted after adjustment for BMI and VAT (17). In type 2 diabetes, ultrasound studies similarly linked greater para- or perirenal fat thickness with CKD and renal resistance indices (18). These studies established a question worth pursuing, but their cross-sectional design could not determine whether fat accumulation preceded renal dysfunction, resulted from the same metabolic milieu, or increased as renal disease evolved.

The literature in established CKD is similarly informative but heterogeneous. In a CKD cohort, PRAT thickness tracked adverse metabolic characteristics (19). In biopsy-evaluated CKD, renal sinus fat was associated with intrarenal haemodynamic abnormalities independently of visceral fat (20). These findings support anatomical specificity, but selection matters. Biopsy cohorts are enriched for renal disease severe or uncertain enough to justify tissue diagnosis; they cannot be assumed to represent community CKD. Diabetes is another major source of selection. Much of the positive literature comes from type 2 diabetes, a state in which visceral adiposity, insulin resistance, hyperglycaemia, hyperfiltration, and pharmacological treatment are tightly intertwined (21). The evidence is therefore strongest for an adiposity-enriched metabolic CKD setting, not for every CKD aetiology.

### 5.2 Longitudinal renal outcomes

Prospective evidence is smaller but more consequential. In a longitudinal diabetic cohort without CKD at baseline, greater PRAT thickness was associated with incident CKD over two years; the study reported 29 incident events among 190 participants and a hazard ratio of 1.67 per standard-deviation increase in PRAT thickness (22). The temporal sequence is useful, but the event count is too small to support a clinical prediction rule. A separate prospective study of 56 patients with CKD reported that greater renal sinus fat accumulation was associated with subsequent severe eGFR decline (23). This is a prognostic signal, not yet an externally validated biomarker. These small studies are important precisely because they move beyond prevalence, but their precision and generalisability remain limited. Studies directly linking measured kidney-adiposity compartments to kidney failure, cardiovascular events, or mortality remain very limited; most longitudinal depot studies use incident CKD or eGFR decline rather than hard clinical endpoints.

Recent imaging studies add nuance by separating kidney fat compartments. Dixon MRI in people with obesity demonstrated that renal parenchymal fat, renal sinus fat, and PRAT can be measured simultaneously and that their associations with renal function are not identical (16). CT studies in diabetic kidney disease have likewise begun to distinguish fat quantity from attenuation, suggesting that a depot's imaging characteristics may matter as much as its size (14). These studies are a step toward multidimensional characterisation rather than simple thickness measurement. They still rely mainly on contemporaneous renal endpoints, however, and they do not show that a more elaborate imaging description improves clinical decisions.

### 5.3 Incremental value beyond established CKD markers

The most persuasive reason to continue studying kidney-specific adiposity is not that its association with CKD reaches statistical significance. Many correlated markers can do that. The clinically relevant question is incremental information. A kidney-adiposity measure would need to improve prediction or treatment decisions beyond established variables such as age, diabetes, blood pressure, eGFR, albuminuria, waist measures, and, where available, VAT. Some studies report persistence of associations after BMI or VAT adjustment (17, 20, 22), but formal evaluation of calibration, discrimination, reclassification, and decision utility is uncommon. The 2025 meta-analysis of perirenal fat and CKD included only eight studies and 2,576 participants, underscoring both the reproducibility of the signal and the limited size of the evidence base (7). Accordingly, the available evidence supports a narrow conclusion: kidney-associated adiposity carries renal information in selected populations, but its incremental clinical value has not been established. A recent CT study in 404 participants with type 2 diabetes directly examined incremental prognostic value: perirenal adipose-tissue attenuation predicted rapid DKD progression among the 125 participants with kidney-function follow-up (24). This is a relevant proof-of-concept, but the diabetes-only sample, limited longitudinal subset, and absence of external validation or decision-curve evidence preclude a claim of routine clinical utility. Future studies should therefore benchmark kidney-adiposity measures against validated CKD risk models rather than rely only on statistical independence from BMI or VAT.

## 6 Mechanism: from anatomical proximity to human biological evidence

Mechanistic arguments in this field are often stronger than the human data that support them. Anatomical proximity makes several pathways plausible. Expansion of fat within the renal sinus could influence low-pressure venous and lymphatic structures, alter interstitial pressure, and contribute to intrarenal haemodynamic changes. PRAT can supply fatty acids, adipokines, cytokines, and neurohumoral signals to a kidney already exposed to systemic obesity-related stress. Intrarenal lipid can reflect an imbalance between fatty-acid uptake, oxidation, and export. Each pathway is biologically credible, but credibility should not be confused with proof of a continuous causal chain from depot expansion to CKD progression (3, 6).

Renal physiology provides one of the better human bridges. In 51 people with type 2 diabetes, Spit and colleagues combined MRI-based renal sinus fat assessment with measured GFR, effective renal plasma flow, and renal vascular resistance. Greater renal sinus fat was associated with lower measured filtration and plasma flow and with higher renal vascular resistance; several associations persisted after adjustment for general adiposity (25). In a separate study of 91 people with type 2 diabetes, greater PRAT was associated with renal vascular and afferent arteriolar resistance, and statistical mediation analysis suggested that these haemodynamic variables accounted for part of the association with GFR (26). Because the exposure, mediator, and outcome were assessed in an observational framework and afferent resistance was model-derived, the analysis supports haemodynamic plausibility and quantifies statistical mediation under model assumptions but does not establish a causal pathway.

Human tissue now provides a second bridge. The paired donor study by Pereira and colleagues is particularly valuable because PRAT and RSAT were sampled from the same individuals, limiting between-person confounding when the two depots were compared. The marked differences in oxidative, thermogenic, macrophage, and inflammatory signatures show that anatomical proximity does not imply biological equivalence (12). This finding changes how imaging data should be interpreted. A larger PRAT layer and a larger RSAT volume cannot automatically be read as two measurements of the same inflammatory process. The local biological state may differ even when the depots enlarge in parallel. What remains missing is comparable paired tissue in people with obesity and CKD across disease stages, linked to imaging and renal physiology.

Systemic endocrine and inflammatory pathways remain relevant, but they should occupy a supporting rather than dominant role in kidney-adiposity research. Leptin, adiponectin, RAAS activation, sympathetic signalling, macrophage recruitment, oxidative stress, and inflammatory cytokines provide plausible links between adipose dysfunction and renal injury (3, 6). These mechanisms explain why VAT and dysfunctional adipose tissue matter even if kidney-adjacent fat proves to be only a marker. They also create a confounding problem: a PRAT measure may correlate with renal injury because it is a regional index of systemic visceral adiposity. Mechanistic studies therefore need to determine whether local kidney-associated depots exert effects that are separable from the endocrine and haemodynamic consequences of whole-body metabolic disease.

Intrarenal lipid introduces a different biological question. A higher renal fat fraction does not necessarily imply adipocyte expansion; it may reflect lipid droplets within renal cells and a mismatch between substrate delivery and mitochondrial oxidation. Dixon MRI studies show that renal fat can be quantified and is associated with early kidney abnormalities in obesity and diabetes (16). Small randomized imaging studies further suggest that intrarenal lipid can change with pharmacological treatment, which is discussed below. The limitation is tissue attribution: imaging-derived fat fraction does not directly identify the cell type, lipid species, or toxic intermediate responsible for injury. Human biopsy or molecular studies are needed to connect the imaging profile to lipotoxic stress rather than neutral lipid storage.

Emerging functional genomics offers another line of evidence. Multi-omics work has identified adipose-to-kidney signalling candidates, including an oncostatin M-OSMR axis linked to renal fibrotic pathways (27). Such studies are useful because they move beyond generic statements about circulating cytokines and nominate traceable ligand-receptor relationships. They do not establish that a single pathway dominates obesity-associated CKD, and they are not yet a basis for clinical characterisation. Their greater value is methodological: future kidney-adiposity studies can combine imaging-defined depots with tissue or circulating molecular readouts to ask whether a specific biological state, rather than volume alone, predicts renal response.

Taken together, the mechanistic literature supports a working model in which location, tissue state, and host susceptibility modify the effect of adipose burden. It does not yet close the causal chain. The human evidence identified here does not demonstrate the full sequence in which expansion of a defined kidney-associated depot precedes a defined local biological change, that change produces progressive renal injury, and selective reversal of the depot or pathway prevents that injury. This distinction is central to the therapeutic question.

## 7 Treatment-associated changes in kidney adiposity and the unresolved causal link

Treatment studies address three distinct outcomes: loss of body weight or VAT, reduction of a kidney-associated fat compartment, and improvement in kidney disease. Representative human studies directly assessing treatment-associated kidney-adiposity remodeling and mechanistic trial frameworks are compiled in Table 3. These outcomes are related but not interchangeable. Contemporary obesity and CKD therapies can improve renal outcomes through haemodynamic, metabolic, anti-inflammatory, or other mechanisms that do not require measurable reduction of PRAT or RSAT. Figure 3 therefore separates global adiposity, kidney-associated adiposity, and kidney disease into parallel evidence streams.

**Table 3:** Studies directly measuring treatment-associated kidney-adiposity change and mechanistic trial designs.

Study/intervention	Population/design	Kidney-depot measure	Depot finding	Renal outcome finding	Mediation tested?	Interpretation
Zelicha 2018—lifestyle weight loss	*n* = 278; randomized 18-mo diet/lifestyle trial	MRI RSF + renal parenchymal fat	RSF decreased ∼9%; parenchymal fat little change	RSF change not associated with renal-function or BP improvement	No	Depot remodeling is possible; renal mediation not shown
Spurny 2022—dietary weight loss	*n* = 137; longitudinal imaging analysis	MRI kidney sinus/cortical fat	Sinus fat decreased in greater weight-loss strata; cortical fat did not respond similarly	No causal renal-outcome bridge	No	Renal compartments respond differently to weight loss
Zhang 2023—canagliflozin	*n* = 64 newly diagnosed T2D; randomized vs. glimepiride; 24 wk	MRI renal fat fractio*n* + oxygenation	Renal fat fraction fell; oxygenation indices improved	No CKD clinical-outcome mediation	No	Randomized intrarenal imaging signal; not CKD mediation
Cuatrecasas 2024—dapagliflozin + metformin	*n* = 29 T2D + obesity; open-label randomized; 6 mo	US perirenal fat layer	Greater perirenal fat loss than metformin alone	Kidney outcome was not the tested mediator endpoint	No	Direct PRAT remodeling signal in a small metabolic cohort
Lin 2024—glucose-regulation interventions	T2D; *n* = 93; retrospective analysis of intervention imaging data	MRI RSF	RSF changes differed by ethnicity; liraglutide contrast was not significant after adjustment	No renal mediation endpoint	No	Illustrates heterogeneity and a null adjusted treatment contrast
ADIPO-CKD 2026—SGLT2i exposure	CKD stages 1–4; *n* = 189; prospective observational; 8 mo	US PRAT thickness	PRAT decreased in the SGLT2i group	Renal/inflammatory changes did not establish a causal bridge	No	CKD-specific remodeling association; nonrandomized
Moriconi 2026—bariatric surgery	Severe obesity; postoperative re-imaging *n* = 50	MRI RSF/RSF%	Postoperative RSF change tracked anthropometric change	Renal mediation not established	No	Direct surgical RSF-remodeling signal; observational subgroup
REMODEL 2025—semaglutide	T2D + CKD; randomized mechanism-of-action trial; design/baseline publication	Multimodal kidney MRI + biopsy	Design supports serial mechanistic assessment; definitive depot-mediation result not established in this publication	Kidney mechanistic endpoints incorporated	Planned/not established by design paper	Directly relevant mechanistic trial; current peer-reviewed report is design/baseline
TRANSCEND-CKD 2026—retatrutide	Overweight/obesity + CKD; *n* = 146; phase 2b RCT design/baseline publication	MRI kidney-associated fat planned	No efficacy result yet	Primary planned endpoint: change in iohexol-measured GFR	Planned/not yet reported	Design capable of testing the missing bridge; no efficacy claim

The main table is restricted to studies with direct kidney-adiposity measurement or mechanistic trial designs that incorporate such measurement. Large kidney-outcome trials without kidney-depot assessment are discussed only as therapeutic context and are mapped in Supplementary Table S1. Kidney outcome benefit and treatment-associated depot remodeling are separate observations unless linked within the same temporally ordered mediation analysis. RSAT denotes the anatomical renal-sinus adipose depot; RSF is retained when used as the imaging term in the source study.

**Figure 3:**
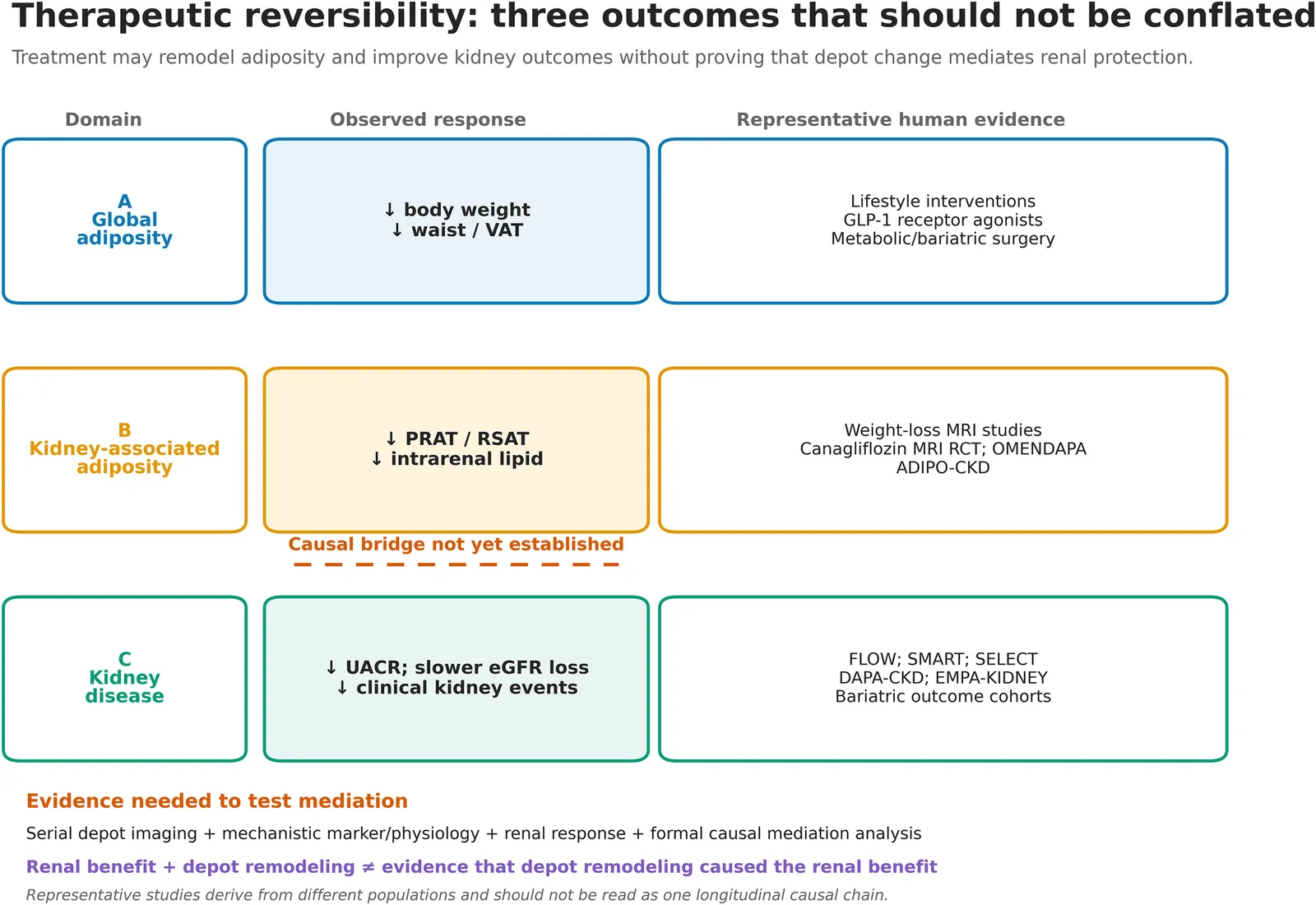
Treatment-associated change should be separated into global adiposity, kidney-associated adiposity, and kidney disease. Selected interventions can reduce body fat or alter kidney-associated adiposity. Large outcome trials and surgical cohorts demonstrate kidney benefit in relevant populations but generally did not measure kidney-adiposity compartments and are therefore contextual rather than depot-specific evidence. These parallel observations do not establish that kidney-depot remodeling caused the renal benefit. Representative studies derive from different populations and should not be interpreted as one longitudinal causal chain. Testing mediation requires serial depot imaging, a mechanistically coherent intermediate readout, a renal response, and a formal causal mediation analysis.

First, kidney-associated fat is modifiable. In an 18-month randomized lifestyle trial of 278 participants with abdominal obesity or dyslipidaemia, renal sinus fat fell by approximately 9% overall, whereas renal parenchymal fat changed little. Renal sinus fat loss tracked weight loss, but the change was not associated with improvement in renal function or blood pressure (28). A later dietary imaging study similarly showed substantial reductions in kidney sinus fat among participants who achieved greater weight loss, while cortical fat did not show the same response (29). These studies provide a useful negative lesson: anatomical remodeling can be demonstrated without establishing renal functional mediation.

SGLT2 inhibition provides more direct pharmacological evidence of kidney-fat remodeling. In a small randomized trial of 64 patients with newly diagnosed type 2 diabetes and normal renal function, canagliflozin reduced MRI-derived renal fat fraction over 24 weeks and improved cortical and medullary oxygenation indices; change in fat fraction correlated with change in cortical oxygenation (30). In the open-label OMENDAPA trial, 29 drug-naive patients with type 2 diabetes and obesity were randomized to metformin or metformin plus dapagliflozin in the context of a hypocaloric diet. The combination group had greater perirenal fat-layer reduction over six months (31). These are intervention-linked imaging signals, but neither trial establishes that the observed depot change explains prevention of CKD progression.

The ADIPO-CKD study moves the remodeling question into established CKD. In a prospective observational cohort of 189 patients with CKD stages 1–4, 50 patients treated with an SGLT2 inhibitor had a reduction in ultrasound-measured PRAT from 1.28 ± 0.70 to 0.91 ± 0.61 cm over eight months (change −0.37 cm), whereas the non-SGLT2i group did not show the same pattern. Associations remained after adjustment that included BMI and visceral-fat change (32). The study is important because it demonstrates longitudinal kidney-adjacent adipose remodeling in CKD. Its design also limits causal inference: treatment allocation was not randomized, the treated group was small, and renal-function, albuminuria, and inflammatory changes did not establish a formal mediation pathway. The data support an association between SGLT2i exposure and PRAT remodeling; they do not establish that PRAT reduction mediates renoprotection.

Large kidney-outcome trials of SGLT2 inhibitors establish renal benefit but did not measure PRAT, RSAT, or intrarenal lipid longitudinally. DAPA-CKD and EMPA-KIDNEY are therefore retained as therapeutic context, not as evidence that kidney-adiposity remodeling mediates benefit (33, 34).

The same distinction applies to semaglutide. FLOW, SMART, and SELECT established kidney or albuminuria benefits in relevant metabolic and CKD populations but did not measure kidney-adiposity compartments (35–37). They therefore cannot determine whether depot change contributed to the renal effect. A prespecified SMART body-composition analysis quantified total fat and lean mass rather than kidney-specific adiposity (33), reinforcing this limitation. REMODEL is more directly relevant to this review because its mechanism-of-action design in people with type 2 diabetes and CKD incorporates multimodal kidney MRI and biopsy; the peer-reviewed publication currently establishes the design and baseline framework rather than definitive kidney-adiposity mediation (38).

Global weight loss cannot be assumed to substitute for kidney-depot remodeling. Different renal fat compartments respond differently to weight loss (28, 29), and an MRI analysis of glucose-regulation interventions did not demonstrate a robust liraglutide-specific reduction in renal sinus fat after adjustment (34). GLP-1 receptor agonism may alter kidney-associated adiposity in some settings, but the existing renal-outcome evidence does not require that mechanism. Demonstrating mediation would require serial kidney-depot measurement within a trial that also captures renal response, with temporal ordering and a formal mediation analysis planned within the study design.

Metabolic surgery provides a useful contrast. MRI follow-up shows that renal sinus fat can remodel after bariatric surgery (37), whereas larger surgical cohorts provide long-term kidney-outcome evidence without serial kidney-depot measurement (39–41). Those outcome studies are therefore contextual rather than depot-specific evidence, and the contribution of renal-sinus-fat change to postoperative kidney benefit remains uncertain.

Demonstrating a causal treatment–depot–kidney link would require a more demanding sequence: a treatment must change a standardised kidney-adiposity measure; the depot change should precede and track a biologically coherent local or physiological response; that response should relate to a renal endpoint; and a protocol-specified mediation or treatment-effect analysis should support a role beyond changes in systemic adiposity. TRANSCEND-CKD is designed to address part of this gap. The phase 2b trial randomised 146 participants with overweight or obesity and CKD to retatrutide or placebo and incorporates iohexol-measured GFR and MRI-based kidney measures, including kidney-associated fat (35). The current publication reports rationale, design, and baseline characteristics rather than efficacy. At present, TRANSCEND-CKD informs the design of mechanistic trials; it does not provide evidence that retatrutide reverses kidney-adiposity states.

## 8 Discussion: from kidney-adiposity measurement to clinical utility

Kidney-adjacent fat can now be quantified with reasonable technical precision, but measurement alone does not create a clinically useful measure. This is especially relevant when cut points are proposed. A PRAT thickness or RSAT volume that separates groups within one cohort does not automatically become a clinical threshold because values depend on modality, anatomical plane, sex, body size, metabolic context, and case mix. The studies identified here do not support a sufficiently standardised and externally validated PRAT, RSAT, renal-fat-fraction, or composite kidney-adiposity threshold for CKD diagnosis or treatment (11, 13).

Independence from BMI or VAT is also insufficient to establish clinical usefulness. Nephrologists already have strong predictors of progression, including eGFR, albuminuria, diabetes, blood pressure, age, cardiovascular disease, and validated kidney-risk models. A kidney-associated depot measure matters only if it improves prediction or changes a decision after those variables and systemic adiposity have been considered. Opportunistic assessment from abdominal CT or MRI obtained for another indication is probably the most realistic near-term setting because it requires no additional procedure. Automated segmentation may make such workflows scalable, but technical success must be followed by CKD-specific external validation and proof that any added prediction is clinically meaningful. Even then, a significant regression coefficient is not enough: nested model comparisons, calibration, discrimination, reclassification where appropriate, and decision-analytic evaluation are needed before routine reporting can be justified.

For now, kidney-adiposity measurements are more useful as investigative variables than as diagnostic tools. Systemic adiposity describes metabolic background; PRAT, RSAT, and intrarenal lipid describe anatomically distinct renal exposures; tissue or imaging characteristics may add biological information; and renal measurements indicate whether those exposures are organ-relevant. Reproducible depot measures that add to established prediction would strengthen the clinical case, but treatment-effect modification would be more compelling. If a kidney-adiposity measure identified patients with differential response to SGLT2 inhibition, GLP-1 receptor agonism, metabolic surgery, or another intervention, it could inform decisions rather than merely describe anatomy. The human studies identified here have not yet established such treatment-effect modification.

Interpretation is constrained by inconsistent nomenclature and the close correlation between regional and systemic adiposity. PRAT, perinephric fat, para-perirenal fat, renal sinus fat, and generic ‘renal fat’ are used differently across disciplines, and some ultrasound protocols include layers that CT or MRI studies segment separately. PRAT and RSAT also commonly expand alongside VAT. Adjustment for VAT is useful but cannot remove measurement error, residual confounding, or shared biology. Visceral adiposity itself is plausibly relevant to CKD, with Mendelian-randomization evidence supporting that relationship (4). Regional measures therefore need to show what they add rather than reproduce the systemic adiposity pattern.

Generalisability is limited by the populations in which most positive studies were performed. Type 2 diabetes contributes a large share of the human evidence, where hyperglycaemia, microvascular disease, visceral adiposity, hyperfiltration, and medication effects are tightly linked. These cohorts are clinically relevant but represent a metabolically enriched setting rather than the full obesity–CKD interface. SMART is informative because semaglutide reduced albuminuria in overweight or obese CKD patients without diabetes (36), yet kidney-adiposity depots were not measured. Prospective kidney-adiposity assessment in non-diabetic obesity-associated CKD remains limited. Healthy kidney-donor studies provide unusually clean anatomical and tissue comparisons but little information about disease behaviour (12).

Reverse causation is difficult to exclude once CKD is established. CKD can alter physical activity, appetite, insulin metabolism, adipose inflammation, fluid balance, and muscle mass (2); a fat measurement obtained after disease onset may therefore reflect both exposure and consequence. Mechanistic endpoints require similar caution. Statistical mediation in cross-sectional haemodynamic data is not causal mediation, circulating inflammatory markers do not establish local adipose-to-kidney signalling, and an MRI fat fraction does not identify a toxic lipid species. Repeated measurements, temporal ordering, and tissue- or pathway-specific validation are needed before these links can be treated as causal in humans.

Progress depends first on measurement standardisation rather than adding further indices. PRAT and RSAT need shared anatomical definitions and minimum reporting standards across ultrasound, CT, and MRI, including laterality, kidney size, body size, VAT adjustment, and inter-reader reliability. Prospective multicentre cohorts should then test incremental information rather than repeat cross-sectional correlations. Useful designs would combine systemic adiposity, kidney-specific depots, UACR, measured or standardised estimated GFR, and longitudinal outcomes, with external validation across sex, age, ethnicity, diabetes status, and CKD stage. Paired imaging–tissue studies in obesity and CKD are also needed to test whether attenuation, fat fraction, or related imaging characteristics correspond to adipocyte dysfunction, immune composition, fibrosis, or lipid species. Automated pipelines should be reported with external segmentation performance, failure rates, human quality-control procedures, and robustness across scanners and acquisition protocols before they are used for clinical risk assessment.

Intervention trials provide the most direct setting in which to test whether a modifiable depot contributes to renal response. Serial depot imaging should be embedded in trials of treatments that alter body weight or kidney outcomes, with depot measurements preceding renal endpoints whenever possible. The intermediate readout should match the mechanism: measured GFR, renal plasma flow, or validated perfusion imaging for haemodynamic hypotheses, and tissue- or pathway-specific markers for inflammatory hypotheses. Formal causal mediation requires explicit assumptions, temporal ordering, adequate sample size, and sensitivity analyses; correlation between change scores is insufficient. TRANSCEND-CKD is well positioned to address part of this question because kidney imaging and iohexol-measured GFR are built into the trial (35); its current publication reports design and baseline characteristics, not efficacy.

Routine clinical adoption would require more than causal plausibility. Even a causal depot is not automatically worth measuring. The measure would need to change monitoring, prognosis, or treatment selection enough to justify acquisition, segmentation, and reporting. Until such evidence is available, kidney-adiposity measurements are most useful for defining anatomy precisely, testing what regional adiposity adds beyond systemic adiposity, and separating treatment-associated remodeling from mediation of kidney benefit.

## 9 Conclusion

Kidney-associated adiposity is not a single anatomical or biological entity. Human imaging studies associate PRAT, RSAT, and intrarenal lipid with renal measures, and selected prospective cohorts suggest that these measures can carry prognostic information beyond a contemporaneous cross-sectional association. Human physiology and tissue studies add biological specificity, but their scope matters: in healthy kidney donors, PRAT and RSAT show distinct tissue programs, while selected clinical cohorts link kidney-associated fat to intrarenal haemodynamics (12, 25). The current literature does not establish a validated clinical classification, a universal depot threshold, or a kidney-adiposity measure that should guide treatment selection. Much of this evidence comes from type 2 diabetes-enriched cohorts, so generalisation to non-diabetic obesity-associated CKD remains uncertain.

Intervention evidence remains split across two lines of research. Selected human studies show that lifestyle-induced weight loss and SGLT2 inhibition can alter kidney-associated fat measures (28, 30–32), and postoperative imaging suggests renal-sinus-fat remodeling after metabolic surgery (37). Separately, randomized trials show renal benefit with SGLT2 inhibitors and semaglutide (36, 39, 40, 42), while observational surgical cohorts associate metabolic surgery with favorable long-term kidney outcomes (41). Across the human studies identified in this review, formal evidence that depot change mediates renal benefit remains lacking. Closing that gap is more important than demonstrating another parallel association.

At present, compartment-resolved assessment of kidney adiposity is best regarded as a research approach rather than a clinical scoring system. Its value will depend on anatomical standardisation, evidence that imaging measures correspond to meaningful biology, prospective incremental information beyond established CKD predictors and validated risk models, and trials that directly test whether depot modification contributes to kidney response. Until those conditions are met, kidney-adiposity measures can refine biological questions and interpretation, but they should not determine treatment choice.
