# Supplementary material for: Kidney adiposity in obesity and chronic kidney disease: mechanisms, measurement, and clinical implications
Source: Front Med (Lausanne). 2026 Oct 22;13:1967640. doi: 10.3389/fmed.2026.1967640 (PMC13735895; doi:10.3389/fmed.2026.1967640)
Supplement: Supplementary file 1 [file Table1.docx]

Supplementary Table S1. Human evidence map for kidney-adiposity research

Scope. This study-level evidence map lists the human studies used to structure the narrative synthesis. It is a transparent narrative-review evidence map rather than an exhaustive systematic-review dataset. Sample sizes were rechecked against the primary PubMed record and, where needed, the corresponding full-text report. “Not applicable” is used only when a single participant count is not meaningful for a multi-dataset functional-genomic analysis. N denotes the total analytic cohort unless otherwise specified; validation, longitudinal, randomized, or subgroup sample sizes are shown separately when they differ from the overall cohort.

Evidence contribution terminology. Labels map directly to Figure 2: Measurement validity / anatomical definition (prerequisite), Association, Partial independence from systemic adiposity, Prospective association, Human mechanistic support, Treatment-associated depot remodeling, Context outside the depot continuum: kidney-outcome evidence without depot measurement, and Planned mechanistic bridge. No included study is classified as establishing causal mediation or treatment-effect modification for a kidney-adiposity measure. Studies were selected to represent the most informative human evidence across compartments, modalities, populations, and claim types; selection was not based on statistical significance, and this evidence map is not a formal risk-of-bias assessment.

**Terminology.** PRAT denotes perirenal adipose tissue; RSAT denotes the anatomical renal sinus adipose depot. RSF is retained when the source study uses renal sinus fat as an imaging measure. Study-specific cut points are not treated as universal clinical thresholds.

S1A. Measurement validity and anatomical characterisation

| **Study / reference** | **Population (N)** | **Design** | **Depot / measure** | **Modality** | **Kidney / related endpoint** | **Principal finding** | **Evidence contribution** | **Key limitation** |
| --- | --- | --- | --- | --- | --- | --- | --- | --- |
| Favre et al., 2017 [S1-01] PMID: 28423008 | Overweight/obesity (N=40) | Cross-sectional validation study | PRAT / RSF | CT | Not a renal outcome study | PRFT correlated more strongly with perirenal fat volume than renal-sinus fat area. | Measurement validity / anatomical definition | Small validation cohort; no kidney outcomes. |
| de Boer et al., 2021 [S1-02] PMID: 32297700 | Healthy adults >40 years (N=19) | Test-retest imaging study | RSF and renal MRI parameters | MRI | Imaging repeatability | Renal-sinus fat volume/percentage showed good repeatability, with ICC values reported up to ~0.98. | Measurement validity / anatomical definition | Technical study; limited clinical inference. |
| Kawasaki et al., 2010 [S1-03] PMID: 20806348 | Fatty-liver/metabolic assessment cohort (N=286) | Cross-sectional imaging study | Para-/perirenal fat | Ultrasound | Metabolic/fatty-liver profile | Shows feasibility of ultrasound-based para/perirenal fat measurement in metabolic characterisation. | Measurement validity / anatomical definition | Not designed for CKD outcomes. |
| Kawasaki et al., 2008 [S1-04] PMID: 18027837 | Body-fat assessment cohort (N=57) | Cross-sectional validation study | Para-/perirenal fat | Ultrasound + CT comparator | Visceral adiposity | Ultrasound para/perirenal fat correlated with CT visceral fat; a study-specific ≥10 mm marker was proposed for visceral fat deposition. | Measurement validity / anatomical definition | Historical, non-CKD study; study-specific cutoff. |
| Gong et al., 2007 [S1-05] PMID: 17392130 | Metabolic syndrome (N=72) | Imaging comparison study | Visceral/abdominal fat including perirenal approaches | Ultrasound / MRI | Metabolic profile | Supports non-invasive ultrasound as a pragmatic but less comprehensive alternative to MRI for visceral-fat assessment. | Measurement validity / anatomical definition | Not designed for renal adiposity/CKD. |
| Pimanov et al., 2020 [S1-06] PMID: 32857464 | Visceral-fat imaging cohort (N=304 overall; CT-correlation subset n=104; metabolic-syndrome cut-off subset n=200) | Cross-sectional imaging validation | Visceral / para-perirenal fat | Ultrasound + CT | Metabolic syndrome | Different ultrasound visceral-fat locations correlate with CT measures, but thresholds are context-specific. | Measurement validity / anatomical definition | Metabolic-syndrome rather than CKD population. |
| Caglar et al., 2014 [S1-07] PMID: 25242158 | Normal subjects (N=240) | Cross-sectional anatomical study | RSF | CT | Anatomical quantification | Demonstrates CT-based volumetric quantification of renal sinus fat in normal subjects. | Measurement validity / anatomical definition | Normal-subject anatomical study. |
| Abdeldayem et al., 2022 [S1-08] PMID: 36304908 | Hypertension/control cohort (N=80; 40/40) | Cross-sectional CT study | RSF | MDCT | Hypertension | RSF surface area can be quantified and is associated with hypertension/BMI in the studied cohort. | Association | Hypertension-focused; no CKD endpoint. |
| Notohamiprodjo et al., 2020 [S1-09] PMID: 32074103 | Glycemic-state cohort (N=366; normal n=230 / prediabetes n=87 / diabetes n=49) | Cross-sectional MRI study | Renal fat / RSF | MRI | Metabolic profile | Shows that renal and renal-sinus fat can be quantified across glycemic states and may vary with metabolic status. | Association | Cross-sectional; not CKD-focused. |
| Gjela et al., 2022 [S1-10] PMID: 35849166 | Obesity/lean comparison (N=42; 28/14) | Method comparison study | Renal fat / RSF | MRI | Imaging quantification | Demonstrates method-dependent quantification of renal fat in obesity. | Measurement validity / anatomical definition | Methodological study. |
| Yokoo et al., 2016 [S1-11] PMID: 27007212 | T2D/non-T2D comparison (N=69; 29/40) | Cross-sectional MRI study | Renal parenchymal lipid | Dixon MRI | Renal steatosis / diabetes profile | Supports Dixon-based MRI as a non-invasive method for renal steatosis quantification in T2D. | Measurement validity / anatomical definition | Cross-sectional and diabetes-specific. |
| Aydın et al., 2024 [S1-12] PMID: 37341174 | CKD imaging cohort (N=88; CKD-d n=42 / CKD-nd n=31 / controls n=15) | Cross-sectional MRI study | Renal parenchymal lipid | Chemical-shift MRI | CKD imaging profile | Supports feasibility of assessing regional renal parenchymal steatosis in CKD. | Association | Cross-sectional; protocol dependence. |
| Tang et al., 2024 [S1-13] PMID: 38839650 | Obesity/control imaging cohort (N=103; 56/47) | Prospective/cross-sectional imaging cohort | Renal PDFF / RSF volume / PRFT | Dixon MRI | eGFR / early kidney damage | Multiple kidney-fat compartments were quantifiable; PRFT remained negatively associated with eGFR after adjustment including BMI, VAT, SAT and other renal-fat measures. | Partial independence from systemic adiposity | Modest sample; limited clinical endpoints. |
| Alhulail et al., 2022 [S1-14] PMID: 35629890 | Healthy volunteers (N=5) | Technical feasibility/reliability study | Intrarenal lipid | 3T MRSI | Imaging reliability | Demonstrates feasibility and reliability of spectroscopic renal lipid quantification. | Measurement validity / anatomical definition | Technical study; limited outcome data. |
| Li et al., 2025 [S1-15] PMID: 41381068 | Metabolic syndrome/control cohort (N=45; 24/21) | Cross-sectional MRI study | Renal fat fraction | MRI | Subclinical renal injury | MRI fat fraction and diffusion measures identify renal changes associated with metabolic syndrome. | Association | Small sample; not CKD outcome study. |
| Liu et al., 2025 [S1-16] PMID: 40619382 | T2D/control MRI cohort (N=131; 105/26) | Cross-sectional MRI study | Renal fat | MRI fat + oxygenation parameters | DKD severity | Links renal fat deposition with renal hypoxia imaging patterns in DKD. | Human mechanistic support | Diabetes-specific; cross-sectional. |
| Liu et al., 2025 [S1-17] PMID: 40287889 | T2D/control MRI cohort (N=107; 81/26) | Cross-sectional MRI study | Renal ectopic fat | MRI | Renal haemodynamics | Associates renal ectopic fat with haemodynamic abnormalities in T2D. | Human mechanistic support | Cross-sectional / diabetes-specific. |
| Hua et al., 2024 [S1-18] PMID: 38951835 | T2D biomarker-imaging cohort (N=133) | Cross-sectional biomarker-imaging study | Renal fat | MRI + biomarkers | Metabolic/renal profile | Provides associative biomarker evidence linking metabolic stress pathways to renal fat content. | Human mechanistic support | Cross-sectional and biomarker-based. |
| Wang et al., 2018 [S1-19] PMID: 29450711 | Early diabetic nephropathy/control cohort (N=95; 61/34) | Cross-sectional MRI study | Renal fat fraction | MRI / DTI | Early renal injury | Shows combined renal fat and diffusion abnormalities in early DKD. | Association | Small/diabetes-specific imaging study. |
| Białek et al., 2025 [S1-20] PMID: 40869714 | CKD/control CT cohort (N=237; 117/120) | Retrospective CT study | PRAT + kidney volume | CT | CKD status | Suggests routine CT may support opportunistic CKD characterisation using kidney volume and perirenal fat measures. | Association | Retrospective, modest sample, needs external validation. |
| Peng et al., 2026 [S1-21] PMID: 41076326 | Controls and T2D cohort (N=730; controls n=151; T2D n=579 [non-DKD n=281 / DKD n=298]) | Retrospective CT study | Renal / PRAT / RSF | CT | DKD status | Quantity and quality measures of kidney-related fat differ across T2D/DKD strata, with RSF area and attenuation providing descriptive information. | Association | Retrospective single-disease context. |
| Moriconi et al., 2026 [S1-22] PMID: 41390907 | Severe obesity/lean controls (N=121; 74/47); postoperative re-imaging n=50 | MRI cohort with postoperative repeat imaging | RSF | MRI | eGFR / RSF | RSF/RSF% related to anthropometric measures and eGFR; postoperative RSF change tracked changes in WHtR/BRI in the re-imaged subgroup. | Treatment-associated depot remodeling | Observational postoperative subgroup; no formal analysis linked RSF change to a kidney outcome or tested mediation. |
| Li et al., 2024 [S1-64] PMID: 38573065 | Clear-cell RCC; multicentre N=614 | Multicentre retrospective technical study | PRAT extracted after automated kidney/VAT segmentation | CT + deep learning | Automated PRAT morphology/feature extraction | Demonstrates technical feasibility of automated segmentation and PRAT feature extraction in renal imaging. | Measurement validity / technical feasibility | Oncology population; not CKD validation; clinical risk utility for CKD not established. |

S1B. Association, independence and prospective outcome studies

| **Study / reference** | **Population (N)** | **Design** | **Depot / measure** | **Modality** | **Kidney / related endpoint** | **Principal finding** | **Evidence contribution** | **Key limitation** |
| --- | --- | --- | --- | --- | --- | --- | --- | --- |
| Foster et al., 2011 [S1-23] PMID: 21931075 | Community cohort (N=2,923) | Community cross-sectional cohort | RSF | CT | Hypertension / CKD | Fatty kidney was associated with hypertension and CKD; associations persisted after BMI/VAT adjustment, including CKD OR ~1.86 after adjustment. | Association | Cross-sectional renal outcomes. |
| Lamacchia et al., 2011 [S1-24] PMID: 20798120 | T2D (N=151) | Cross-sectional clinical study | Para-/perirenal fat | Ultrasound | CKD / renal resistance index / uric acid | Greater para-/perirenal fat thickness was independently associated with CKD and higher renal resistance index in T2D. | Association | Cross-sectional, diabetes-specific. |
| Geraci et al., 2018 [S1-25] PMID: 30218482 | Hypertension cohort (N=296) | Cross-sectional cohort | Para-/perirenal fat | Ultrasound | eGFR | Perirenal fat was associated with lower eGFR after accounting for other adiposity indices. | Partial independence from systemic adiposity | Cross-sectional hypertensive population. |
| D'Marco et al., 2019 [S1-26] PMID: 31357262 | Established CKD (N=103) | Cross-sectional CKD cohort | PRAT | Ultrasound | Metabolic/renal risk profile | PRFT was associated with an adverse metabolic risk profile in CKD. | Association | Cross-sectional; potential residual confounding. |
| Fang et al., 2020 [S1-27] PMID: 32685560 | T2D (N=171) | Cross-sectional clinical study | PRAT | Ultrasound | Reduced eGFR | Greater PRFT was associated with reduced GFR in T2D. | Association | Cross-sectional; diabetes-specific. |
| Shen et al., 2020 [S1-28] PMID: 32653293 | T2D (N=89; albuminuria n=66 / no albuminuria n=23) | Cross-sectional clinical study | PRAT | Ultrasound | Urine albumin excretion | PRFT was positively associated with albuminuria in T2D. | Association | Cross-sectional; diabetes-specific. |
| Chen et al., 2021 [S1-29] PMID: 34593536 | T2D without baseline CKD (longitudinal n=190; incident CKD n=29) | Longitudinal cohort | PRAT | Ultrasound/clinical imaging | Incident CKD over 2 years | Per-SD PRFT was associated with incident CKD (HR 1.67, 95% CI 1.04–2.68); reported discrimination exceeded VAT in this cohort. | Prospective association | Only 29 CKD events; diabetes-specific; limited external validation. |
| Hu et al., 2022 [S1-30] PMID: 36507087 | T2D (cross-sectional N=959; albuminuria-free longitudinal n=218) | Cross-sectional + longitudinal cohort | PRAT | Ultrasound | Albuminuria onset/progression | In longitudinal analysis, greater PFT was associated with earlier albuminuria development (reported HR ~2.83). | Prospective association | Study-specific cutoff/short follow-up; diabetes-specific. |
| Shen et al., 2022 [S1-31] PMID: 36246918 | T2D (N=189) | Cross-sectional MRI study | Renal fat fraction | MRI | CKD | Higher renal fat fraction was associated with CKD; highest-tertile association and AUC were reported in the study. | Association | Cross-sectional; diabetes-specific; modest precision. |
| Fujioka et al., 2023 [S1-32] PMID: 37095344 | Biopsy-selected CKD (N=56) | Prospective biopsy-selected CKD cohort | RSF | CT/MRI-based RSF quantification | Future >50% eGFR decline | Greater RSF accumulation was associated with adverse renal prognosis in this small CKD cohort. | Prospective association | Very small biopsy-selected cohort; limited generalizability. |
| Xu et al., 2023 [S1-33] PMID: 37883495 | T2D (N=337) | Cross-sectional study | Para-/perirenal fat | Ultrasound | Reduced eGFR / CKD | Perirenal thickness was associated with reduced GFR and showed moderate discrimination (reported AUC ~0.686). | Partial independence from systemic adiposity | Cross-sectional; modest discrimination; diabetes-specific. |
| Kaneko et al., 2024 [S1-34] PMID: 38555192 | Biopsy-evaluated CKD (N=132) | Cross-sectional biopsy CKD cohort | RSF | CT/imaging + renal indices/histology | eGFR / renal resistance / histology | RSF volume was associated with intrarenal haemodynamic abnormalities independent of visceral fat in CKD. | Human mechanistic support | Cross-sectional biopsy-selected cohort. |
| Kono et al., 2024 [S1-35] PMID: 38471672 | CKD with/without diabetes (N=473; non-DM n=202 / T2D n=271) | Cross-sectional CKD study | PRAT | Imaging | Renal dysfunction | The PRAT–renal dysfunction relationship appeared stronger in participants with diabetes than without diabetes. | Association | Cross-sectional; population-specific; interaction requires cautious interpretation. |
| Tang et al., 2024 [S1-36] PMID: 39219990 | T2D undergoing coronary catheterization (N=603; contrast-induced nephropathy n=77) | Observational cohort | PRAT | Imaging | Contrast-induced nephropathy | Greater PRFT was associated with contrast-associated kidney injury in this selected T2D population. | Association | Selected procedural population; observational. |
| Cho et al., 2023 [S1-37] PMID: 36818142 | Acute decompensated heart failure (N=266) | Observational cohort | PRAT | Imaging | Kidney function deterioration | PRFT was independently associated with reduced/worsening kidney function in ADHF. | Association | Heart-failure confounding; acute setting. |
| Boorsma et al., 2023 [S1-38] PMID: 37656080 | HFpEF/control cohort (N=192; HFpEF n=94 / controls n=98) | Observational physiological cohort | PRAT | Imaging + haemodynamic assessment | Renal dysfunction / haemodynamics | PRAT was associated with renal dysfunction and abnormal haemodynamics in HFpEF. | Human mechanistic support | HFpEF-specific confounding and selection. |
| Kamat et al., 2026 [S1-39] PMID: 42149464 | Predialysis CKD/control cohort (N=90; DKD n=30 / non-DKD CKD n=30 / controls n=30) | Cross-sectional CKD study | PRAT / EAT | Ultrasound + biomarkers | Inflammation / oxidative stress / metabolic risk | Provides contemporary associative links between PRAT, inflammatory/oxidative biomarkers and CKD profile. | Association | Cross-sectional; systemic biomarkers cannot establish local mediation. |
| Kukla et al., 2026 [S1-40] PMID: 41853746 | Living kidney donors (N=3,226) | Large donor cohort with CT and implantation biopsy | Posterior PRAT | CT + implantation biopsy | Long-term eGFR <45 and histologic correlates | Posterior PRAT was associated with histologic changes and long-term lower kidney function, whereas BMI was less informative in the reported analysis. | Human mechanistic support | Healthy-donor selection limits generalisability to CKD populations. |
| Xu et al., 2026 [S1-41] PMID: 41087204 | High-normal blood pressure cohort (N=166) | Prospective cohort | PRAT | Imaging | Incident hypertension / albuminuria-related mediation | Greater PRFT was associated with incident hypertension; the study reported statistical mediation by albumin-creatinine ratio. | Prospective association | Small cohort; outcome is hypertension, mediation is statistical. |
| Fan et al., 2025 [S1-63] PMID: 40480065 | T2D (N=404; kidney-function follow-up n=125) | Retrospective CT cohort with longitudinal subset | Perirenal adipose tissue area/index + attenuation | CT | KDIGO risk category / rapid DKD progression | Perirenal adipose-tissue attenuation predicted rapid DKD progression in the longitudinal subset and provided incremental prognostic information. | Prospective / incremental association | Diabetes-specific; only 125 with follow-up; no external validation or decision-impact analysis. |

S1C. Human mechanistic support

| **Study / reference** | **Population (N)** | **Design** | **Depot / measure** | **Modality** | **Kidney / related endpoint** | **Principal finding** | **Evidence contribution** | **Key limitation** |
| --- | --- | --- | --- | --- | --- | --- | --- | --- |
| Spit et al., 2020 [S1-42] PMID: 31471702 | T2D physiological cohort (N=51) | Cross-sectional physiological study | RSF | MRI + inulin/PAH-based physiology | Measured GFR / ERPF / renal vascular resistance | Greater RSF was associated with lower measured GFR/ERPF and higher renal vascular resistance; several associations persisted after adiposity adjustment. | Human mechanistic support | Small cross-sectional T2D cohort. |
| Chen et al., 2024 [S1-43] PMID: 39389218 | T2D physiological cohort (N=91) | Cross-sectional physiological study with statistical mediation | PRAT | MRI/clinical imaging + GFR/ERPF + Gomez-derived arteriolar resistance | GFR / renal vascular and arteriolar resistance | Renal vascular and afferent arteriolar resistance statistically mediated part of the PRAT–GFR association (reported ~29% and ~41%). | Human mechanistic support | Cross-sectional, model-derived arteriolar resistance, diabetes-specific. |
| Pereira et al., 2025 [S1-44] PMID: 40696281 | Healthy kidney donors (N=40 paired depots; RNA-seq n=30) | Paired human adipose-tissue functional/transcriptomic study | PRAT vs RSAT | Biopsy, adipocyte assays, RNA-seq, immunohistochemistry | Tissue metabolic/inflammatory profile | PRAT showed greater thermogenic/oxidative signatures and insulin-stimulated glucose uptake, whereas RSAT was more macrophage/inflammation enriched; 1113 DEGs were reported. | Human mechanistic support | Healthy donors; cross-sectional tissue biology; disease generalizability limited. |
| Zhang et al., 2025 [S1-45] PMID: 40451900 | Multiple functional-genomic and genetic datasets; no single aggregate participant N applicable | Multi-omics / functional genomics | Adipose-derived molecular signals | Functional genomics / omics | Kidney fibrosis pathways | Provides a genetically/functionally traceable adipose-to-kidney signalling hypothesis involving OSM–OSMR. | Human mechanistic support | Multi-omics inference; requires disease-specific prospective validation. |
| Boissier et al., 2020 [S1-46] PMID: 32256495 | Deceased kidney donors (N=53; optimal n=22 / expanded-criteria n=31) | Human donor adipose-tissue study | PRAT | Adipose stromal-vascular tissue / immune profiling | Early graft dysfunction | Shows an inflammatory PRAT signature in older/marginal donors and association with early graft dysfunction. | Human mechanistic support | Transplant/donor context; not native CKD. |

S1D. Treatment-associated remodeling, kidney-outcome context and planned bridge studies

| **Study / reference** | **Population (N)** | **Design** | **Depot / measure** | **Modality** | **Kidney / related endpoint** | **Principal finding** | **Evidence contribution** | **Key limitation** |
| --- | --- | --- | --- | --- | --- | --- | --- | --- |
| Zhang et al., 2023 [S1-47] PMID: 38057876 | Newly diagnosed T2D with normal renal function (N=64; canagliflozin n=33 / glimepiride n=31) | Randomized controlled trial | Intrarenal lipid | Functional MRI | Renal fat fraction / renal oxygenation | Canagliflozin reduced renal fat fraction (reported 1.79±0.61 to 1.32±0.62) and improved cortical/medullary oxygenation indices; change in fat fraction correlated with change in cortical R2*. | Treatment-associated depot remodeling | Small T2D cohort with normal renal function; no CKD outcomes/formal mediation. |
| Cuatrecasas et al., 2024 [S1-48] PMID: 38734755 | Drug-naive T2D with obesity (N=29; combination n=15 / metformin n=14) | Open-label randomized phase IV trial | PRAT | Ultrasound | Perirenal fat layer | Combination treatment produced greater perirenal fat loss than metformin alone over 6 months; weight loss also occurred. | Treatment-associated depot remodeling | Very small open-label trial; obesity/T2D rather than CKD; kidney outcomes not primary. |
| Zelicha et al., 2018 [S1-49] PMID: 28501343 | Abdominal obesity/dyslipidemia (N=278 randomized) | Randomized dietary/lifestyle trial | RSF / renal parenchymal fat | MRI | RSF / renal parenchymal fat; renal function/BP | RSF decreased by about 9% overall; renal parenchymal fat change was smaller/non-significant; RSF change tracked weight loss but was not associated with improved renal function or blood pressure. | Treatment-associated depot remodeling | Not a CKD trial; renal outcomes not primary; treatment arms complex. |
| Spurny et al., 2022 [S1-50] PMID: 35406052 | Overweight/obesity (N=137) | Post-hoc dietary intervention imaging study | Kidney sinus fat / cortical fat | MRI | Kidney-fat change | Kidney-sinus fat decreased substantially in the higher weight-loss strata at weeks 12 and 50, while cortical fat did not show the same pattern. | Treatment-associated depot remodeling | Post-hoc; not CKD-focused; stratified by achieved weight loss. |
| Lin et al., 2024 [S1-51] PMID: 38085104 | T2D (N=93) | Retrospective analysis of intervention imaging data | RSF | MRI | RSF change | RSF changes differed by ethnicity; liraglutide did not show a significant treatment contrast versus placebo after adjustment in the reported subgroup analyses. | Treatment-associated depot remodeling | Retrospective/subgroup analysis; modest sample; treatment contrast non-significant. |
| Checa-Ros et al., 2026 [S1-52] PMID: 42194603 | CKD stages 1-4 (N=189; SGLT2i n=50 / no SGLT2i n=139) | Prospective observational cohort | PRAT / EAT | Ultrasound | PRAT/EAT; renal function/UACR/inflammation | PRAT decreased from 1.28±0.70 to 0.91±0.61 cm in the SGLT2i group (Δ−0.37 cm); renal-function and systemic-inflammatory improvements were trends rather than definitive significant mediation evidence. | Treatment-associated depot remodeling | Observational treatment allocation; small treated group; no formal causal mediation. |
| Perkovic et al., 2024 [S1-53] PMID: 38785209 | T2D + CKD (N=3,533; semaglutide n=1,767 / placebo n=1,766) | Randomized double-blind placebo-controlled trial | No kidney depot measured | Clinical outcomes | Major kidney disease composite / eGFR slope | Primary events 331 vs 410; HR 0.76 (95% CI 0.66–0.88); median follow-up 3.4 y; eGFR slope and CV/all-cause outcomes favored semaglutide. | Context outside the depot continuum: kidney-outcome evidence without depot measurement | No kidney-depot imaging/tissue mediator in the trial. |
| Apperloo et al., 2025 [S1-54] PMID: 39455729 | Overweight/obesity + CKD without diabetes (N=101; semaglutide n=51 / placebo n=50) | Randomized double-blind placebo-controlled trial | No kidney depot measured | Clinical outcomes | UACR; GFR/eGFR measures | Semaglutide reduced UACR by 52.1% versus placebo at 24 weeks; short-term measured/estimated GFR improvement was not demonstrated. | Context outside the depot continuum: kidney-outcome evidence without depot measurement | Small, short-duration trial; albuminuria endpoint; no kidney-depot imaging. |
| Heerspink et al., 2026 [S1-55] PMID: 42308057 | SMART trial analysis (N=101) | Prespecified randomized-trial analysis | Whole-body composition, not kidney depot | Body composition + GFR | Fat/lean mass; measured/estimated GFR | Semaglutide reduced fat and lean mass, but changes in body composition did not correlate with measured or estimated GFR changes. | Context outside the depot continuum: kidney-outcome evidence without depot measurement | Secondary analysis; no PRAT/RSF measurement. |
| Colhoun et al., 2024 [S1-56] PMID: 38796653 | Overweight/obesity + established CVD without diabetes (N=17,604; semaglutide n=8,803 / placebo n=8,801) | Prespecified trial analysis | No kidney depot measured | Clinical outcomes | Kidney composite / eGFR | Kidney composite occurred in 1.8% vs 2.2% (HR 0.78, 95% CI 0.63–0.96); eGFR differences favored semaglutide. | Context outside the depot continuum: kidney-outcome evidence without depot measurement | Population selected for CVD rather than CKD; no depot measurement. |
| Heerspink et al., 2020 [S1-57] PMID: 32970396 | CKD with/without T2D (N=4,304; dapagliflozin n=2,152 / placebo n=2,152) | Randomized double-blind placebo-controlled trial | No kidney depot measured | Clinical outcomes | ≥50% eGFR decline / ESKD / renal or CV death | Primary outcome 197 vs 312; HR 0.61 (95% CI 0.51–0.72); median follow-up 2.4 y. | Context outside the depot continuum: kidney-outcome evidence without depot measurement | No kidney-depot imaging/tissue measurement. |
| EMPA-KIDNEY Collaborative Group, 2023 [S1-58] PMID: 36331190 | Broad CKD with/without diabetes (N=6,609; empagliflozin n=3,304 / placebo n=3,305) | Randomized double-blind placebo-controlled trial | No kidney depot measured | Clinical outcomes | Kidney disease progression or CV death | Primary outcome 432/3304 vs 558/3305; HR 0.72 (95% CI 0.64–0.82); median follow-up ~2.0 y. | Context outside the depot continuum: kidney-outcome evidence without depot measurement | No kidney-depot measurement. |
| Shulman et al., 2018 [S1-59] PMID: 29568103 | Severe obesity (N=4,047; surgery n=2,010 / usual care n=2,037) | Prospective controlled intervention cohort | No kidney depot measured | Clinical outcomes | ESRD / CKD stage 4 or ESRD | Over median 18 y, ESRD occurred in 13 vs 26; adjusted HR 0.27 (95% CI 0.12–0.60). | Context outside the depot continuum: kidney-outcome evidence without depot measurement | Nonrandomized surgical allocation; historical procedures; no depot measure. |
| Sørensen et al., 2025 [S1-60] PMID: 40804715 | Bariatric surgery/matched obesity cohort (surgery n=18,827; matched controls n=94,135) | Population-based matched cohort | No kidney depot measured | Registry outcomes | AKI / nephrolithiasis / CKD / KFRT | Median 8.1 y; surgery associated with lower CKD (aHR 0.41) and KFRT (aHR 0.63), but higher AKI (aHR 1.63) and nephrolithiasis (aHR 1.73). | Context outside the depot continuum: kidney-outcome evidence without depot measurement | Observational residual confounding; no kidney-depot measure. |
| Lee et al., 2025 [S1-61] PMID: 40875559 | CKD + severe obesity (N=1,538; surgery n=563 / nonsurgical n=975) | Population-based cohort | No kidney depot measured | Linked administrative/clinical data | Mortality / major adverse kidney events | Median follow-up 7.7 y; bariatric surgery associated with lower mortality (HR 0.48) and MAKE (HR 0.47). | Context outside the depot continuum: kidney-outcome evidence without depot measurement | Observational allocation; no depot measurement. |
| Heerspink et al., 2026 [S1-62] PMID: 41160422 | Overweight/obesity + CKD (N=146 randomized; 367 screened) | Phase 2b randomized double-blind placebo-controlled mechanistic trial (design/baseline paper) | PRAT / RSF planned alongside kidney structure/function | MRI + iohexol mGFR | Iohexol mGFR; kidney structure/hemodynamics; kidney-associated fat | The trial is designed to test mechanistic kidney effects and includes kidney-adiposity imaging, but the publication used here reports design/baseline characteristics, not efficacy results. | Planned mechanistic bridge | No treatment-outcome results in the design/baseline paper. |
| Cherney et al., 2025 [S1-65] PMID: 40608494 | T2D + CKD; randomized mechanism-of-action trial | Randomized trial design/baseline publication | Kidney adiposity and parenchymal measures | Multimodal kidney MRI + biopsy | Mechanistic kidney endpoints | REMODEL incorporates serial kidney MRI and biopsy to test semaglutide mechanisms in T2D+CKD; the peer-reviewed report establishes design/baseline rather than definitive depot mediation. | Planned mechanistic bridge | Design publication; should not be interpreted as efficacy or mediation evidence. |

**Abbreviations:** BMI, body mass index; CKD, chronic kidney disease; CT, computed tomography; DKD, diabetic kidney disease; eGFR, estimated glomerular filtration rate; ERPF, effective renal plasma flow; GFR, glomerular filtration rate; MRI, magnetic resonance imaging; MRS/MRSI, magnetic resonance spectroscopy/spectroscopic imaging; PRAT, perirenal adipose tissue; RSAT, renal sinus adipose tissue; RSF, renal sinus fat; T2D, type 2 diabetes; UACR, urinary albumin-to-creatinine ratio; VAT, visceral adipose tissue.

References for Supplementary Table S1

Each study listed in Supplementary Table S1 is linked one-to-one to the reference identifier shown in the first column. PubMed identifiers are included to facilitate verification.

S1-01. Guillaume Favre, Caroline Grangeon-Chapon, Charles Raffaelli, Florence François-Chalmin, Antonio Iannelli, Vincent Esnault. Perirenal fat thickness measured with computed tomography is a reliable estimate of perirenal fat mass. PLoS One. 2017;12(4):e0175561. doi:10.1371/journal.pone.0175561. PMID:28423008.

S1-02. Anneloes de Boer, Anita A Harteveld, Bjorn Stemkens, Peter J Blankestijn, Clemens Bos, Suzanne L Franklin, et al. Multiparametric Renal MRI: An Intrasubject Test-Retest Repeatability Study. J Magn Reson Imaging. 2021;53(3):859-873. doi:10.1002/jmri.27167. PMID:32297700.

S1-03. Satsuki Kawasaki, Osamu Hasegawa, Shinobu Satoh, Kazushi Numata, Yasuo Terauchi. Sonographic assessment of fatty liver infiltration using measurement of para- and perirenal fat thickness. J Clin Ultrasound. 2010. doi:10.1002/jcu.20736. PMID:20806348.

S1-04. Satsuki Kawasaki, Kazutaka Aoki, Osamu Hasegawa, Kazushi Numata, Katsuaki Tanaka, Naomi Shibata, et al. Sonographic evaluation of visceral fat by measuring para- and perirenal fat. J Clin Ultrasound. 2008. doi:10.1002/jcu.20426. PMID:18027837.

S1-05. Weikun Gong, Huilong Ren, Hongzhang Tong, Xiaoyin Shen, Jianping Luo, Shuting Chen, et al. A comparison of ultrasound and MRI to assess visceral fat in metabolic syndrome. Asia Pac J Clin Nutr. 2007. PMID:17392130.

S1-06. Sergey Pimanov, Vladimir Bondarenko, Elena Makarenko. Visceral fat in different locations assessed by ultrasound: Correlation with computed tomography and cut-off values in patients with metabolic syndrome. Clin Obes. 2020;10(6):e12404. doi:10.1111/cob.12404. PMID:32857464.

S1-07. V Caglar, A Songur, M Acar, R Uygur, O A Alkoc, T Acar. Volumetric evaluation of fat in the renal sinus in normal subjects using stereological method on computed tomography images. Folia Morphol. 2014. doi:10.5603/FM.2014.0016. PMID:25242158.

S1-08. Emad H Abdeldayem, Mohamed G Mansour, Basant M Raief Mosaad. Measurement of the Surface Area of the Renal Sinus Fat Using MDCT: Correlation with Presence and Severity of Essential Hypertension and Body Mass Index. J Belg Soc Radiol. 2022;106(1):91. doi:10.5334/jbsr.2776. PMID:36304908.

S1-09. Mike Notohamiprodjo, Martin Goepfert, Susanne Will, Roberto Lorbeer, Fritz Schick, Wolfgang Rathmann, et al. Renal and renal sinus fat volumes as quantified by magnetic resonance imaging in subjects with prediabetes, diabetes, and normal glucose tolerance. PLoS One. 2020. doi:10.1371/journal.pone.0216635. PMID:32074103.

S1-10. Mimoza Gjela, Anders Askeland, Jens Brøndum Frøkjær, Maiken Mellergaard, Aase Handberg. MRI-based quantification of renal fat in obese individuals using different image analysis approaches. Abdom Radiol. 2022. doi:10.1007/s00261-022-03603-4. PMID:35849166.

S1-11. Takeshi Yokoo, Haley R Clark, Ivan Pedrosa, Qing Yuan, Ivan Dimitrov, Yue Zhang, et al. Quantification of renal steatosis in type II diabetes mellitus using Dixon-based MRI. J Magn Reson Imaging. 2016. doi:10.1002/jmri.25252. PMID:27007212.

S1-12. Hüseyin Aydın, Hasan Aydın, Adnan Karaibrahimoğlu, Baris Afsar. Evaluation of the parenchymal distribution of renal steatosis in chronic kidney disease using chemical shift MRI. Adv Clin Exp Med. 2024. doi:10.17219/acem/166512. PMID:37341174.

S1-13. H Tang, L Xie, L Liu, Y Shen, P Yang, J Wu, et al. Renal fat deposition measured on Dixon-based MRI is significantly associated with early kidney damage in obesity. Abdom Radiol (NY). 2024;49(10):3476-3484. doi:10.1007/s00261-024-04391-9. PMID:38839650.

S1-14. Ahmad A Alhulail, Mahsa Servati, Nathan Ooms, Oguz Akin, Alp Dincer, M Albert Thomas, et al. In Vivo Renal Lipid Quantification by Accelerated Magnetic Resonance Spectroscopic Imaging at 3T: Feasibility and Reliability Study. Metabolites. 2022;12(5):386. doi:10.3390/metabo12050386. PMID:35629890.

S1-15. Shisi Li, Yanjun Chen, Yingjie Mei, Xianfu Mo, Jialing Chen, Chijian Li, et al. Noninvasive assessment of metabolic syndrome-related renal injury using MRI fat fraction and apparent diffusion coefficient. J Int Med Res. 2025. doi:10.1177/03000605251399039. PMID:41381068.

S1-16. Jian Liu, Daoyu Yang, Chong Tian, Rui Lv, Rongpin Wang, Xianchun Zeng. Magnetic resonance imaging for noninvasive assessment of renal fat deposition and hypoxia in diabetic kidney disease. BMC Med Imaging. 2025. doi:10.1186/s12880-025-01816-9. PMID:40619382.

S1-17. Jian Liu, Hengzhi Chen, Chong Tian, Liwei Fu, Lisha Nie, Rongpin Wang, et al. Renal ectopic fat deposition and hemodynamics in type 2 diabetes mellitus assessment with magnetic resonance imaging. Insights Imaging. 2025;16(1):93. doi:10.1186/s13244-025-01971-1. PMID:40287889.

S1-18. Yulin Hua, Zaifei Yin, Mingming Li, Hong Sun, Bimin Shi. Correlation between circulating advanced glycation end products and TXNIP levels and renal fat content in type 2 diabetes mellitus. Diabetol Metab Syndr. 2024. doi:10.1186/s13098-024-01361-5. PMID:38951835.

S1-19. Yuan-Cheng Wang, Yinglian Feng, Chun-Qiang Lu, Shenghong Ju. Renal fat fraction and diffusion tensor imaging in patients with early-stage diabetic nephropathy. Eur Radiol. 2018. doi:10.1007/s00330-017-5298-6. PMID:29450711.

S1-20. Piotr Białek, Michał Żuberek, Adam Dobek, Krzysztof Falenta, Ilona Kurnatowska, Ludomir Stefańczyk. Opportunistic Detection of Chronic Kidney Disease Using CT-Based Measurements of Kidney Volume and Perirenal Fat. J Clin Med. 2025. doi:10.3390/jcm14165888. PMID:40869714.

S1-21. Xin-Gui Peng, Ying Cui, Jialu Sun, Xin Chen, Zhou Chen, Wenjing Tian, et al. Stratified CT analysis of renal, perirenal and renal sinus fat in type 2 diabetes and diabetic kidney disease. Nutr Metab Cardiovasc Dis. 2026;36(2):104367. doi:10.1016/j.numecd.2025.104367. PMID:41076326.

S1-22. Diego Moriconi, Miikka-Juhani Honka, Ekaterina Saukko, Emilia Moritz, Aino Latva-Rasku, Prince Dadson, et al. Waist-to-height ratio as a non-invasive marker of renal sinus fat: a magnetic resonance imaging-based cohort study. Int J Obes (Lond). 2026;50(3):586-591. doi:10.1038/s41366-025-01974-4. PMID:41390907.

S1-23. Michael C Foster, Shih-Jen Hwang, Sarah A Porter, Joseph M Massaro, Udo Hoffmann, Caroline S Fox. Fatty kidney, hypertension, and chronic kidney disease: the Framingham Heart Study. Hypertension. 2011;58(5):784-790. doi:10.1161/HYPERTENSIONAHA.111.175315. PMID:21931075.

S1-24. O Lamacchia, V Nicastro, D Camarchio, U Valente, R Grisorio, L Gesualdo, et al. Para- and perirenal fat thickness is an independent predictor of chronic kidney disease, increased renal resistance index and hyperuricaemia in type-2 diabetic patients. Nephrol Dial Transplant. 2011;26(3):892-898. doi:10.1093/ndt/gfq522. PMID:20798120.

S1-25. Giulio Geraci, Marta Maria Zammuto, Alessandro Mattina, Luca Zanoli, Calogero Geraci, Antonio Granata, et al. Para-perirenal distribution of body fat is associated with reduced glomerular filtration rate regardless of other adiposity indices in hypertensive patients. J Clin Hypertens. 2018. doi:10.1111/jch.13366. PMID:30218482.

S1-26. L D'Marco, J Salazar, M Cortez, M Salazar, M Wettel, M Lima-Martínez, et al. Perirenal fat thickness is associated with metabolic risk factors in patients with chronic kidney disease. Kidney Res Clin Pract. 2019;38(3):365-372. doi:10.23876/j.krcp.18.0155. PMID:31357262.

S1-27. Yuan Fang, Yuechao Xu, Yuxian Yang, Chang Liu, Dong Zhao, Jing Ke. The Relationship between Perirenal Fat Thickness and Reduced Glomerular Filtration Rate in Patients with Type 2 Diabetes. J Diabetes Res. 2020. doi:10.1155/2020/6076145. PMID:32685560.

S1-28. Feng-Chih Shen, Ben-Chung Cheng, Jung-Fu Chen. Peri-renal fat thickness is positively associated with urine albumin excretion rate in type 2 diabetes. Obes Res Clin Pract. 2020. doi:10.1016/j.orcp.2020.06.006. PMID:32653293.

S1-29. Xiangjun Chen, Yun Mao, Jinbo Hu, Shichao Han, Lilin Gong, Ting Luo, et al. Perirenal Fat Thickness Is Significantly Associated With Risk for Development of Chronic Kidney Disease in Patients With Diabetes. Diabetes. 2021;70(10):2322-2332. doi:10.2337/db20-1031. PMID:34593536.

S1-30. Hongtu Hu, Wei Liang, Zongwei Zhang, Zikang Liu, Fan Chu, Yan Bao, et al. The Utility of Perirenal Fat in Determining the Risk of Onset and Progression of Diabetic Kidney Disease. Int J Endocrinol. 2022. doi:10.1155/2022/2550744. PMID:36507087.

S1-31. Yan Shen, Lianghua Xie, Xiangjun Chen, Lina Mao, Yao Qin, Rui Lan, et al. Renal fat fraction is significantly associated with risk of chronic kidney disease in type 2 diabetes mellitus. Front Endocrinol (Lausanne). 2022. doi:10.3389/fendo.2022.995028. PMID:36246918.

S1-32. H Fujioka, T Koike, T Imamura, K Kakeshita, H Yamazaki, K Kinugawa. Prognostic impact of renal sinus fat accumulation in patients with chronic kidney disease. Clin Exp Nephrol. 2023;27(7):613-621. doi:10.1007/s10157-023-02350-0. PMID:37095344.

S1-33. Sunan Xu, Junqing Ma, Yongze Zheng, Ruichen Ren, Wenting Li, Wei Zhao, et al. Para-perirenal fat thickness is associated with reduced glomerular filtration rate regardless of other obesity-related indicators in type 2 diabetes mellitus. PLoS One. 2023. doi:10.1371/journal.pone.0293464. PMID:37883495.

S1-34. K Kaneko, R Mitsuno, D Kojima, T Azegami, S Kosugi, T Nakamura, et al. Renal sinus fat is associated with intrarenal hemodynamic abnormalities independent of visceral fat in patients with chronic kidney disease. Obes Res Clin Pract. 2024;18(2):118-123. doi:10.1016/j.orcp.2024.03.005. PMID:38555192.

S1-35. Teruyuki Kono, Gulinu Maimaituxun, Hayato Tanabe, Moritake Higa, Haruka Saito, Kenichi Tanaka, et al. Role of perirenal adiposity in renal dysfunction among chronic kidney disease individuals with or without diabetes: a Japanese cross-sectional study. BMJ Open Diabetes Res Care. 2024. doi:10.1136/bmjdrc-2023-003832. PMID:38471672.

S1-36. Xixiang Tang, Jiafu Wang, Yuman Wu, Zhuoshan Huang, Xiaolan Ouyang, Hongxing Wu, et al. Perirenal Fat Thickness Is Associated With Contrast-Induced Nephropathy in Type 2 Diabetic Patients Undergoing Coronary Catheterization. J Diabetes Res. 2024. doi:10.1155/2024/4905669. PMID:39219990.

S1-37. In-Jeong Cho, Jin Wi, Sang-Eun Lee, Dong-Hyeok Kim, Wook Bum Pyun. Perirenal Fat and Kidney Function Deterioration in Acute Decompensated Heart Failure. Int J Heart Fail. 2023. doi:10.36628/ijhf.2022.0029. PMID:36818142.

S1-38. Eva M Boorsma, Hidemi Sorimachi, Jozine M Ter Maaten, Dirk J van Veldhuisen, Kazunori Omote, Naoki Takahashi, et al. Perirenal Adipose Tissue Is Associated With Renal Dysfunction and Abnormal Hemodynamics in Heart Failure With Preserved Ejection Fraction. JACC Heart Fail. 2023;11(10):1463-1465. doi:10.1016/j.jchf.2023.06.027. PMID:37656080.

S1-39. Namesh Kamat, Surya Prakash Thotanolla, Jeeja Jaya Janardhanan, A Y Lakshmi, Aparna R Bitla, V Vanajakshamma, et al. A study of perirenal and epicardial fat thickness, oxidative stress, inflammation, and metabolic risk in pre-dialysis diabetic and non-diabetic chronic kidney disease patients. Int Urol Nephrol. 2026. doi:10.1007/s11255-026-05193-2. PMID:42149464.

S1-40. Aleksandra Kukla, Anum Iqbal, Syed Khooshal Fareeduddin, Petar Veruovic, Hira Tariq, Pooja Budhiraja, et al. Perirenal Adipose Tissue may be an Important Predictor of Adverse Kidney Outcomes in Living Kidney Donors. Kidney Int Rep. 2026. doi:10.1016/j.ekir.2026.106359. PMID:41853746.

S1-41. Suining Xu, Tingting Zheng, Xiyang Li, Danni Wang, Pengjie Gao, Kamila Kamili, et al. Association between perirenal fat thickness and risk of developing hypertension in a high-normal blood pressure population. Nutr Metab Cardiovasc Dis. 2026. doi:10.1016/j.numecd.2025.104292. PMID:41087204.

S1-42. K A Spit, M H A Muskiet, L Tonneijck, M M Smits, M H H Kramer, J A Joles, et al. Renal sinus fat and renal hemodynamics: a cross-sectional analysis. MAGMA. 2020;33(1):73-80. doi:10.1007/s10334-019-00773-z. PMID:31471702.

S1-43. Xiangjun Chen, Yao Qin, Jinbo Hu, Yan Shen, Yun Mao, Lianghua Xie, et al. Perirenal fat and chronic kidney disease in type 2 diabetes: The mediation role of afferent arteriolar resistance. Diabetes Metab. 2024;50(6):101583. doi:10.1016/j.diabet.2024.101583. PMID:39389218.

S1-44. Maria J Pereira, Athina Mathioudaki, Susanne Hetty, Ali Sedigh, Jan W Eriksson, Maria K Svensson. Depot-specific metabolic and inflammatory profiles in perirenal and renal sinus adipose tissue. Mol Med. 2025;31(1):262. doi:10.1186/s10020-025-01323-1. PMID:40696281.

S1-45. J Zhang, Z Liu, S Dong, K Xu, K Wang, L Gong, et al. Functional genomics reveals adipose-kidney crosstalk as contributor to kidney fibrosis via OSM-OSMR pathway. Funct Integr Genomics. 2025;25(1):114. doi:10.1007/s10142-025-01624-y. PMID:40451900.

S1-46. Romain Boissier, Pauline François, Bastien Gondran Tellier, Maité Meunier, Luc Lyonnet, Stephanie Simoncini, et al. Perirenal Adipose Tissue Displays an Age-Dependent Inflammatory Signature Associated With Early Graft Dysfunction of Marginal Kidney Transplants. Front Immunol. 2020. doi:10.3389/fimmu.2020.00445. PMID:32256495.

S1-47. Li Zhang, Tongdan Wang, Yan Kong, Haizhen Sun, Yuling Zhang, Junmei Wang, et al. Sodium-dependent glucose transporter 2 inhibitor alleviates renal lipid deposition and improves renal oxygenation levels in newly diagnosed type 2 diabetes mellitus patients: a randomized controlled trial. Diabetol Metab Syndr. 2023;15(1):256. doi:10.1186/s13098-023-01236-1. PMID:38057876.

S1-48. Guillem Cuatrecasas, Francisco De Cabo, M José Coves, Ioana Patrascioiu, Gerardo Aguilar, Gabriel Cuatrecasas, et al. Dapagliflozin added to metformin reduces perirenal fat layer in type 2 diabetic patients with obesity. Sci Rep. 2024;14(1):10832. doi:10.1038/s41598-024-61590-6. PMID:38734755.

S1-49. Hila Zelicha, Dan Schwarzfuchs, Ilan Shelef, Yftach Gepner, Gal Tsaban, Lilac Tene, et al. Changes of renal sinus fat and renal parenchymal fat during an 18-month randomized weight loss trial. Clin Nutr. 2018;37(4):1145-1153. doi:10.1016/j.clnu.2017.04.007. PMID:28501343.

S1-50. Manuela Spurny, Yixin Jiang, Solomon A Sowah, Tobias Nonnenmacher, Ruth Schübel, Romy Kirsten, et al. Changes in Kidney Fat upon Dietary-Induced Weight Loss. Nutrients. 2022;14(7):1437. doi:10.3390/nu14071437. PMID:35406052.

S1-51. Ling Lin, Ilona A Dekkers, Qian Tao, Elisabeth H M Paiman, Maurice B Bizino, Ingrid M Jazet, et al. MR Assessed Changes of Renal Sinus Fat in Response to Glucose Regulation in West European and South Asian Patients With Type 2 Diabetes. J Magn Reson Imaging. 2024;60(2):729-738. doi:10.1002/jmri.29174. PMID:38085104.

S1-52. Ana Checa-Ros, Óscar Arias, Owahabanun-Joshua Okojie, Pilar Salvador, Luis D'Marco. Depot-Specific Cardiorenal Adipose Remodeling with SGLT2i in Chronic Kidney Disease. J Clin Med. 2026;15(10):3641. doi:10.3390/jcm15103641. PMID:42194603.

S1-53. Vlado Perkovic, Katherine R Tuttle, Peter Rossing, Kenneth W Mahaffey, Johannes F E Mann, George Bakris, et al. Effects of Semaglutide on Chronic Kidney Disease in Patients with Type 2 Diabetes. N Engl J Med. 2024;391(2):109-121. doi:10.1056/NEJMoa2403347. PMID:38785209.

S1-54. Ellen M Apperloo, Jose L Gorriz, Maria Jose Soler, Secundino Cigarrán Guldris, Josep M Cruzado, Maria Jesús Puchades, et al. Semaglutide in patients with overweight or obesity and chronic kidney disease without diabetes: a randomized double-blind placebo-controlled clinical trial. Nat Med. 2025;31(1):278-285. doi:10.1038/s41591-024-03327-6. PMID:39455729.

S1-55. Hiddo J L Heerspink, Maria Soler, Jelle M Beernink, Niels Jongs, Secundino Cigarran, Josep M Cruzado, et al. Effects of Semaglutide on Body Composition and GFR: A Prespecified Analysis of the SMART Trial. Clin J Am Soc Nephrol. 2026. doi:10.2215/CJN.0000001051. PMID:42308057.

S1-56. Helen M Colhoun, Ildiko Lingvay, Paul M Brown, John Deanfield, Kirstine Brown-Frandsen, Steven E Kahn, et al. Long-term kidney outcomes of semaglutide in obesity and cardiovascular disease in the SELECT trial. Nat Med. 2024;30(7):2058-2066. doi:10.1038/s41591-024-03015-5. PMID:38796653.

S1-57. Hiddo J L Heerspink, Bergur V Stefánsson, Ricardo Correa-Rotter, Glenn M Chertow, Tom Greene, Fan-Fan Hou, et al. Dapagliflozin in Patients with Chronic Kidney Disease. N Engl J Med. 2020;383(15):1436-1446. doi:10.1056/NEJMoa2024816. PMID:32970396.

S1-58. The EMPA-KIDNEY Collaborative Group, William G Herrington, Natalie Staplin, Christoph Wanner, Jennifer B Green, Sibylle J Hauske, et al. Empagliflozin in Patients with Chronic Kidney Disease. N Engl J Med. 2023;388(2):117-127. doi:10.1056/NEJMoa2204233. PMID:36331190.

S1-59. A Shulman, M Peltonen, C D Sjöström, J C Andersson-Assarsson, M Taube, K Sjöholm, et al. Incidence of end-stage renal disease following bariatric surgery in the Swedish Obese Subjects Study. Int J Obes (Lond). 2018;42(5):964-973. doi:10.1038/s41366-018-0045-x. PMID:29568103.

S1-60. Christian Goul Sørensen, Simon Kok Jensen, Reimar Wernich Thomsen, Bente Jespersen, Sigrid Bjerge Gribsholt, Christian Fynbo Christiansen. Kidney outcomes after bariatric surgery: a population-based cohort study. BMC Nephrol. 2025;26(1):458. doi:10.1186/s12882-025-04378-8. PMID:40804715.

S1-61. Yung Lee, Francis Nguyen, Sama Anvari, Amber O Molnar, Aristithes G Doumouras, Michael Walsh, et al. Impact of bariatric surgery on patients with chronic kidney disease and severe obesity: a multiple-linked population-based cohort study. Br J Surg. 2025;112(8):znaf168. doi:10.1093/bjs/znaf168. PMID:40875559.

S1-62. Hiddo J L Heerspink, Daniël H van Raalte, Petter Bjornstad, Mathijs C Bunck, Peikun Wu, Ilke Tunali, et al. Rationale, design and baseline characteristics of the TRANSCEND-CKD trial of retatrutide in patients with chronic kidney disease. Nephrol Dial Transplant. 2026;41(6):1058-1068. doi:10.1093/ndt/gfaf230. PMID:41160422.

S1-63. Jinlei Fan, Liping Zuo, Xiaoyun Liu, Yuqing Tang, Min He, Qichao Cheng, et al. Incremental value of CT-based perirenal fat characteristics in the categorization and progression prediction of diabetic kidney disease. J Diabetes Complications. 2025;39(9):109104. doi:10.1016/j.jdiacomp.2025.109104. PMID:40480065.

S1-64. Shichao Li, Ziling Zhou, Mengmeng Gao, Zhouyan Liao, Kangwen He, Weinuo Qu, et al. Incremental value of automatically segmented perirenal adipose tissue for pathological grading of clear cell renal cell carcinoma: a multicenter cohort study. Int J Surg. 2024;110(7):4221-4230. doi:10.1097/JS9.0000000000001358. PMID:38573065.

S1-65. David Z I Cherney, Nicolas Belmar, Petter Bjornstad, Milenta M Chacko, Thomas P Gunnarsson, Jeffrey B Hodgin, et al. Rationale, design and baseline characteristics of REMODEL, a mechanism-of-action trial with semaglutide in people with type 2 diabetes and chronic kidney disease. Nephrol Dial Transplant. 2025;40(11):2182-2192. doi:10.1093/ndt/gfaf114. PMID:40608494.
